# Crowding for faces is determined by visual (not holistic) similarity: Evidence from judgements of eye position

**DOI:** 10.1038/s41598-018-30900-0

**Published:** 2018-08-22

**Authors:** Alexandra V. Kalpadakis-Smith, Valérie Goffaux, John A. Greenwood

**Affiliations:** 1 Experimental Psychology, University College London, London, United Kingdom; 2 Research Institute for Psychological Science, Université Catholique de Louvain, Louvain-la-Neuve, Belgium; 3 Institute of Neuroscience, Université Catholique de Louvain, Louvain-la-Neuve, Belgium; 4 Department of Cognitive Neuroscience, Maastricht University, Maastricht, The Netherlands

## Abstract

Crowding (the disruption of object recognition in clutter) presents the fundamental limitation on peripheral vision. For simple objects, crowding is strong when target/flanker elements are similar and weak when they differ – a selectivity for target-flanker similarity. In contrast, the identification of upright holistically-processed face stimuli is more strongly impaired by upright than inverted flankers, whereas inverted face-targets are impaired by both – a pattern attributed to an additional stage of crowding selective for “holistic similarity” between faces. We propose instead that crowding is selective for target-flanker similarity in all stimuli, but that this selectivity is obscured by task difficulty with inverted face-targets. Using judgements of horizontal eye-position that are minimally affected by inversion, we find that crowding is strong when target-flanker orientations match and weak when they differ for *both* upright and inverted face-targets. By increasing task difficulty, we show that this selectivity for target-flanker similarity is obscured even for upright face-targets. We further demonstrate that this selectivity follows differences in the spatial order of facial features, rather than “holistic similarity” *per se*. There is consequently no need to invoke a distinct stage of holistic crowding for faces – crowding is selective for target-flanker similarity, even with complex stimuli such as faces.

## Introduction

We have all experienced the difficulty of trying to locate a friend in a busy shopping centre or our favourite shirt in a pile of clothes. This task becomes especially hard when the face or item of clothing that we are searching for is located not in our central/foveal vision, but our visual periphery. Although part of this difficulty can be attributed to reductions in peripheral visual acuity^1,2^, peripheral vision is further limited by *crowding* – the curious phenomenon whereby objects that are otherwise readily identifiable in isolation become jumbled and indistinguishable in clutter^3,4^. The strength and spatial scale of crowding^5,6^ has led to it being described as the fundamental limitation on object recognition in peripheral vision^7^.

Crowding disrupts the identification of a range of fundamental visual dimensions, including orientation^6,8–10^, colour^11–13^, size^14^, and motion^13,15^. Crowding is also selective for differences between the target object and surrounding flanker elements along these dimensions. For simple stimuli, crowding is strong when features in the target and flanker elements are similar in these dimensions, and weak when they differ^10,11,13,16–18^. Importantly, this selectivity for target-flanker similarity is typically *symmetric*: a red target surrounded by green flankers will reduce crowding as much as a green target amongst red flankers.

The generality of this selectivity for target-flanker similarity has been questioned by the observation that the crowding of faces is qualitatively different from crowding for simple objects. Louie *et al*.^19^ found that the identification of an upright target face was more strongly disrupted by upright than inverted flankers, whereas the identification of an inverted target face was equally impaired by *both* upright and inverted flankers. This *asymmetry* was attributed to a selectivity for “holistic similarity”, a criterion that is met when both target and flanker faces are upright, and thus processed holistically^20–24^. Cropped images of houses were also found to be equally impaired by upright and inverted house flankers, matching the pattern seen with inverted target faces. From these findings, Louie *et al*.^19^ claim that similarity in orientation modulates crowding *only* when the target is processed holistically and not for objects processed in a part-based or featural manner (i.e. houses and inverted faces). Although it is odd that this latter “featural” pattern of crowding differs from the above studies with simple stimuli^10,11,13,16^ (where a symmetric selectivity for target-flanker similarity is observed), Louie *et al*. concluded that there are distinct crowding mechanisms for holistically processed faces and featurally processed objects. These findings have been further taken to suggest that crowding consists of multiple independent stages in the visual hierarchy^7,19,25–27^.

We propose an alternative explanation. Although the inversion of a face clearly changes its potential for holistic processing^20–23^, it also increases the difficulty with which the identity of that face is recognised. We suggest that this increase in task difficulty could obscure the more general selectivity for target-flanker similarity that is typically observed for crowding. In other words, it may be these variations in task difficulty, and not the presence or lack of holistic processing *per se*, that cause the curious asymmetric selectivity in the crowding of faces. A demonstration of this nature would eliminate the need for additional processing stages to account for the crowding of faces, and suggest that common principles apply for the crowding of both simple and complex objects. Here, we begin by replicating the “holistic similarity” effect, before introducing an eye-judgement task^28^ that allows the measurement of face crowding with matched difficulty across upright and inverted target conditions, as well as the independent manipulation of task difficulty. We argue that there is indeed a common selectivity for target-flanker similarity that determines the crowding of both simple objects and complex elements such as faces.

## Experiment 1

**Figure 1:**
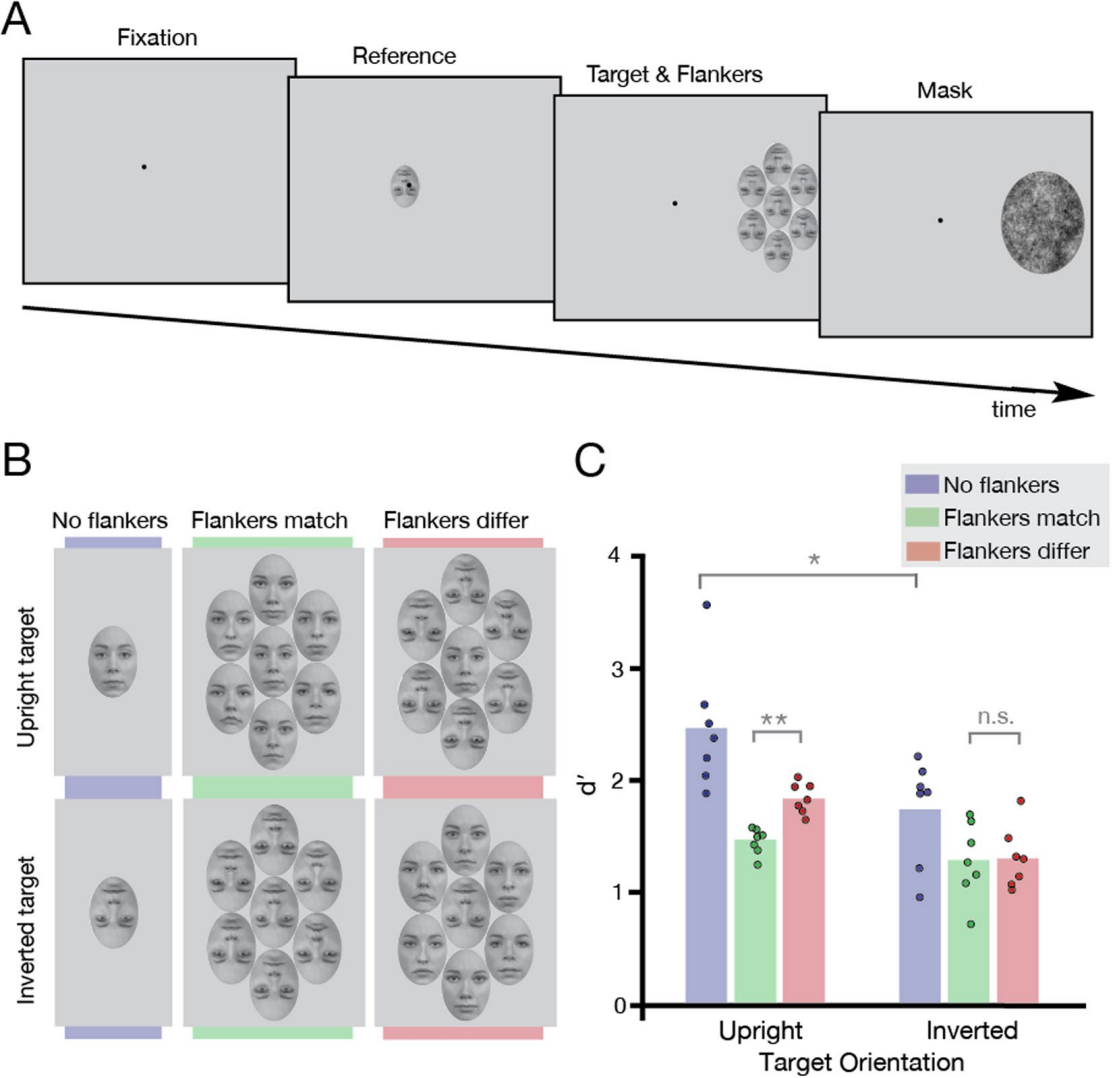
Stimulus time-course, design, and results of the identity-matching procedure in Experiment 1. (**A**) *Stimulus time course:* Each trial began with the presentation of a Gaussian fixation dot, followed by the reference face (500 ms) at fixation, then the target (500 ms, surrounded by flankers) at 12 degrees in the right visual field, and a mask (250 ms). Observers responded after the mask. A flankers-match condition with an inverted target is depicted. This time course was the same for all experiments. (**B**) *Experimental conditions:* Two target orientation conditions were presented in Experiment 1: upright (top panel) and inverted (bottom panel). There were three crowding conditions: the target face presented in isolation (no flankers), the target surrounded by flanker faces of the same orientation (flankers match), and the target surrounded by flankers of different orientation (flankers differ). Face stimuli were taken from the Radboud Faces Database^32^ and edited as described in the Methods. (**C**) *Results of Experiment 1:* Results plotted for the three crowding conditions, for upright and inverted targets (N = 7). Average *d*′ values are depicted by bars; dots show *d*′ for each observer. Grey brackets indicate the significance level of each condition comparison (n.s. not significant, *P < 0.05, **P < 0.01).

Our first aim was to replicate the findings of Louie *et al*.^19^ with some variations in stimuli and experimental design, in order to verify that any conflicting findings were not due to differences in these factors. We thus began with an identity-matching task where observers judged whether a peripheral target face had the same or a different identity as a reference face (Fig. 1A). The target face was presented either upright or inverted, in isolation (“uncrowded”) as well as in the presence of flanker faces (“crowded”) that themselves could be either upright or inverted.

### Methods

#### Observers

Seven observers participated in Experiment 1 (5 females, M_age_ = 23.9 years). This sample size was deemed sufficient given that it doubles that of prior studies^19^. Two of the authors (AKS and JAG) took part in all experiments; the rest of the observers were naïve. All had normal or corrected-to-normal vision. For all experiments, experimental protocols and procedure were approved by the University College London Research Ethics Committee. The protocols and procedure complied with the Declaration of Helsinki, and written informed consent was obtained from all participants prior to each experiment.

#### Apparatus

Experiments were programmed with MATLAB (MathWorks) on a Macintosh computer running PsychToolbox^29–31^. Stimuli were presented on a 21′′ cathode ray tube monitor (LaCie Electron 22 Blue IV) with a resolution of 1152 × 870 pixels and a 75 Hz refresh rate. The monitor was calibrated with a Minolta CS100A photometer to give mean and maximum luminance values of 50 cd/m^2^ and 100 cd/m^2^. Stimuli were viewed binocularly from 50 cm, with head movements minimised using head and chin rests. Experiments took place in a dark room, with responses made using a keyboard.

#### Stimuli

14 adult female Caucasian faces with a neutral frontal gaze were selected from the Radboud Faces Database^32^. Using Adobe Photoshop, all faces were grayscale filtered, and edited to fit their internal features inside an oval window of 272 × 395 pixels. A generic background face texture was created, which was free of external cues like glasses and the hairline that can contribute to identification accuracy^33,34^. The features of each face (eyes, nose, mouth, and chin) were placed onto this matched background, along with a generic pair of eyebrows that was created for all faces. Luminance values were adjusted to span the range from 0–100 cd/m^2^ in all faces. Face RaDF-090-12^32^ was selected as the reference face for all experiments. The remaining 13 faces were used as distractor targets and flankers (Fig. 1B). When flankers were present, six flankers were presented on each trial, fixed on an ellipse around the target with a position along the ellipse that varied from trial to trial (see Fig. 1B).

The size of the faces was determined individually for each observer during practice blocks. By varying the size of face stimuli parametrically we selected a size where observers were capable of at least 90% accuracy with uncrowded upright targets and between 70–80% for targets surrounded by matched flankers. By using such a high performance criterion to determine stimulus size, it is unlikely that any limitations from within-face crowding^35^ (crowding between the features of the face) play a substantial role in our results. Sizes ranged from 2.4–3.6° horizontally and 3.5–5.2° vertically, with means of 3.1° and 4.5°, respectively.

The relative centre-to-centre distance between target and flankers was fixed at 1 × the width of the target. This resulted in variation in the absolute centre-to-centre target-flanker separation between observers, given the above size variations. Nonetheless, if we assume that the region of interference is half the eccentricity of the target, following standard measurements^4,5^, then all of our stimuli fell well within the 6° interference zone for crowding at 12° eccentricity.

#### Procedures

Observers were required to fixate at all times on a Gaussian fixation point with an SD of 3.6 arcmins. Each trial began with the presentation of the reference face at fixation for 500 ms. The location of the reference face was jittered by 25 pixels on each trial to avoid alignment with the peripheral target that could provide extraneous cues to the task, particularly for the eye position judgements in subsequent experiments. This was followed by the presentation of the target face at 12° eccentricity in the right visual field. The target was either presented in isolation or surrounded by 6 flankers. A 1/f noise mask was then presented for 200 ms at the target location (see Fig. 1A), with a size that covered both target and flanker elements for each observer. After presentation of the mask, observers made a same/different judgement for the target face in relation to the reference face, regarding *identity* (“is the target face the same or a different identity from the reference?”). The identity of the target matched that of the reference face on 50% of the trials, and in the rest it was one of the 13 distractor identities.

There were two target orientation conditions, upright and inverted, and three crowding conditions: “uncrowded”, where the target was presented in isolation, “crowded-same”, where the flankers matched the orientation of the target (e.g. upright flankers around an upright target), and “crowded-different”, where the orientation of the flankers differed from the target. This resulted in 2340 trials (3 crowding conditions × 2 same/different conditions × 13 different distractor face identities × 5 repetitions per face identity × 3 blocks × 2 target orientations).

Prior to completing the set of experimental blocks for each experiment, observers completed one or more practice sessions, with auditory feedback for incorrect responses. Practice began with uncrowded trials and progressed to crowded trials after achieving at least 90% accuracy.

### Results and Discussion

We examined the recognition of identity in upright and inverted target faces in three crowding conditions: the target was either uncrowded and presented in isolation, surrounded by flankers of matching orientation, or surrounded by flankers of different orientation (see Fig. 1B). For each condition, *d*′ was calculated as an indicator of face-recognition sensitivity (see Fig. 1C) by subtracting the z-score of the false alarm rate (incorrect “different” responses when target and reference faces were matched) from the z-score of the hit rate (correct “different” responses when target and reference faces differed)^36^. A two-way repeated measures ANOVA was conducted with target orientation and crowding condition as factors. This analysis revealed significant main effects of target orientation (F[1, 6] = 12.84, *P* = 0.012), with identity more accurately recognised in upright than inverted faces, and crowding condition (F[1.15, 6.91] = 19.81, *P* = 0.003, Greenhouse-Geisser corrected). The interaction between target orientation and crowding condition was also significant (F[2, 12] = 9.68, *P* = 0.003). Planned comparisons using paired t-tests with Bonferroni correction showed that for upright target faces, crowding was modulated by the orientation of the flankers: performance was strongly impaired when the upright target was surrounded by similarly upright flankers (“flankers match”) and significantly less impaired with inverted flankers (“flankers differ”; t[6] = −6.91, *P* = 0.0009). Importantly, this was not the case for inverted target faces: both inverted and upright flankers were equally disruptive on performance, with no significant difference between the two (t[6] = 0.27, *P* > 0.99).

We therefore replicate the effects of Louie *et al*.^19^: for an identity-matching task, crowding is modulated by the orientation of the flankers for upright target faces, but not for inverted targets. The lack of selectivity for the orientation of the flankers with inverted target faces has been used to argue that crowding is selective for “holistic similarity”^19,25^. Louie *et al*.^19^ further observed a trend (*P* = 0.055) towards upright flankers being more disruptive on an inverted target than inverted flankers, and speculated that this may be due to holistic information in the upright flankers over-riding the featural information of the inverted targets. Although a subset of our observers also show this pattern, an equal number of observers show the opposite (more crowding with inverted than upright flankers on an inverted target). Therefore, with a larger sample, we find no evidence to support stronger interference with upright flanker faces when the target is inverted.

The basis of the argument for holistic crowding derives from the inversion effect, a difference in the difficulty with which upright and inverted faces are recognised. This difference in difficulty has been taken to suggest distinct processing styles: holistic or configural for upright faces, and featural or part-based for inverted^20–24^. The effect of inversion on face recognition can be seen in the current experiment by comparing upright and inverted uncrowded target face conditions in Fig. 1C. However, this leads to an alternative interpretation of the effects of crowding on faces: we propose that it is the difference in task difficulty with upright vs. inverted target faces that is responsible for the asymmetric pattern of crowding for faces, and not the differential engagement of holistic processing. Namely, the increased difficulty in recognising inverted faces could make it more difficult to then release that target from crowding with flankers of a different orientation, resulting in an apparent lack of target-flanker selectivity. If this were the case, it should be possible to reveal this selectivity for target-flanker similarity with *both* upright and inverted target faces by matching the difficulty of target recognition in upright and inverted conditions.

## Experiment 2

Here we investigated the selectivity of face crowding with matched task difficulty between upright and inverted target orientations. To achieve this, we examined crowding using judgements of the horizontal separation between the eyes (interocular distance) in face stimuli, a task that has been shown to be minimally affected by inversion^28^, and therefore of similar difficulty for upright and inverted targets. For this task, the holistic similarity account^19^ would predict that even with matched difficulty, crowding for inverted target faces should not be modulated by the orientation of the flankers, as inverted faces are not processed holistically. In fact, it could be argued that this eye-judgement task relies primarily on featural rather than holistic processes, given that a significant difference in the difficulty between upright and inverted face recognition is the typical marker of holistic engagement^20,21,23,24^. Were this the case, the holistic account^19^ would predict that there should be no modulation of crowding by the orientation of the flankers for *either* upright or inverted target faces (i.e. the pattern observed above with inverted target faces and houses in an identity-matching task). In contrast, we propose that crowding has a general symmetric selectivity for target-flanker similarity regardless of the degree of holistic engagement, but that this can be obscured by heightened task difficulty. In this case, by matching task difficulty for upright and inverted targets, we should observe selectivity for target-flanker similarity in both target conditions.

### Methods

Methods were generally similar to Experiment 1, with the following exceptions. Seven observers (4 females, M_age_ = 25.1 years) participated in Experiment 2, all with normal or corrected-to-normal vision.

**Figure 2:**
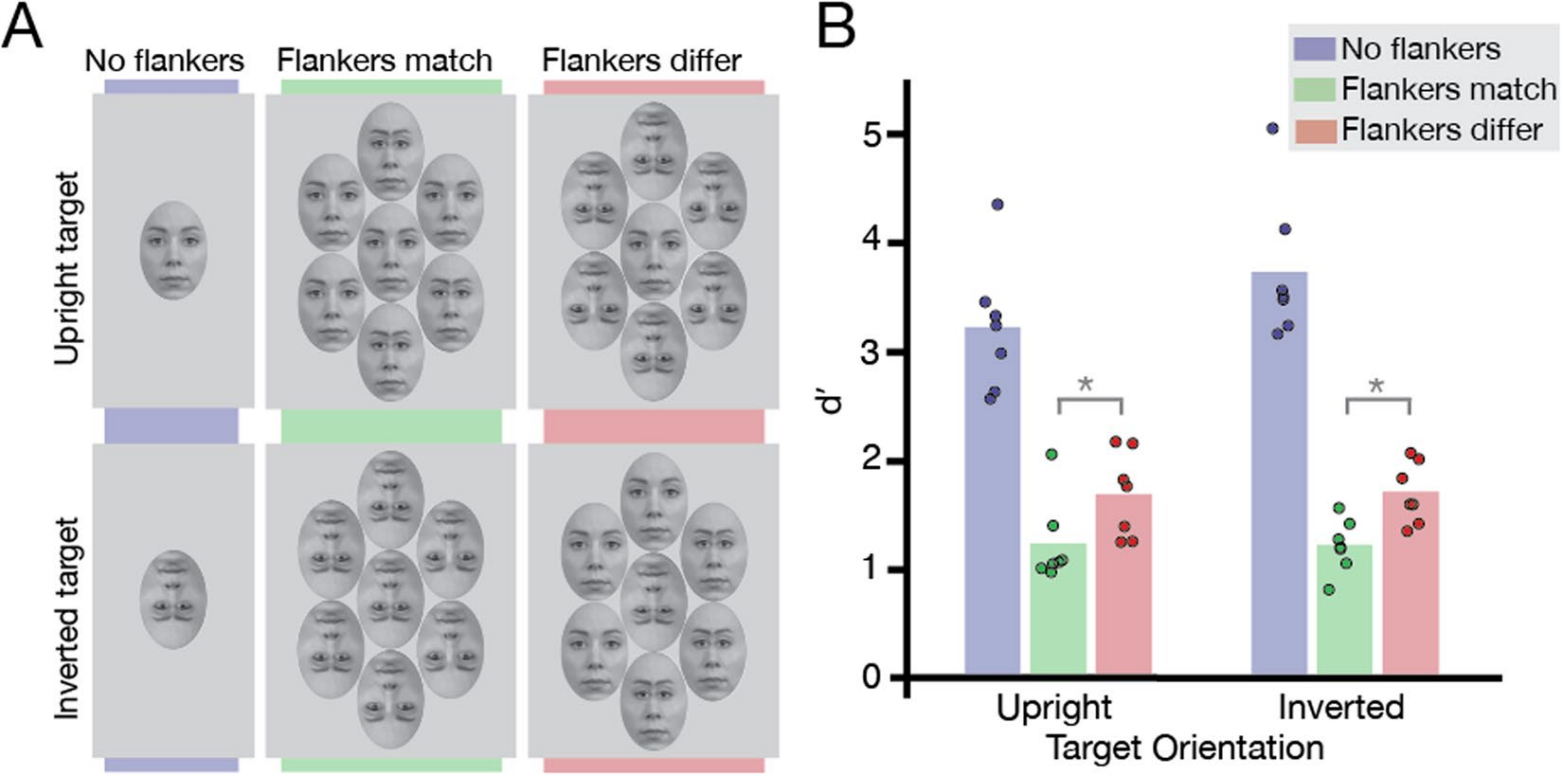
Design and results of the horizontal eye-shift procedure in Experiment 2. (**A**) *Experimental conditions:* Two target orientation conditions were presented in Experiment 2: upright (top panel) and inverted (bottom panel). There were three crowding conditions: the target face presented in isolation (no flankers), the target surrounded by flanker faces of the same orientation (flankers match), and the target surrounded by flankers of different orientation (flankers differ). The horizontal eye position of the target was either the same (“normal”, no eye-shift) or different (eyes shifted inwards). Face stimuli were taken from the Radboud Faces Database^32^ and edited. (**B**) *Results of Experiment 2:* Results plotted for the three crowding conditions, for upright and inverted targets (N = 7). Bars indicate average *d*′; dots indicate *d*′ for each observer. Grey brackets indicate the significance level of each condition comparison (n.s. not significant, *P < 0.05).

To manipulate the horizontal eye positions within faces, the eyes and eyebrows of the reference face were shifted inwards from their original positions within the oval window by 20 pixels. This face with inward-shifted eyes served as the target in the trials when the target differed from the reference face, which was again face RaDF-090-12^32^ with the eyes in their original positions. For the flankers, three had no eye shift (“eyes normal”), while the rest had the eyes shifted 20 pixels inwards from their original positions (see Fig. 2A). Stimulus sizes varied between 2–4° horizontally with a mean of 2.9°, and 2.9–5.8° vertically with a mean of 4.2°. Given these variations in the sizes of the presented faces, this resulted in horizontal eye shifts between 0.15–0.30° for each observer, with a mean of 0.21°.

Observers were shown peripheral target faces at 12° eccentricity with interocular separations that were either unaltered or shifted inwards. They were required to make a same/different judgement for the target in relation to the reference face presented foveally, regarding the *horizontal eye position* (“are the eyes of the target face the same or different from the reference?”). The target had matched eye position to the reference on 50% of the trials, and in the rest it had the eyes shifted inwards.

As in Experiment 1, there were two target orientation conditions, upright and inverted, and three crowding conditions: “uncrowded”, where the target was presented in isolation, “crowded-same”, where the flankers matched the orientation of the target (e.g. upright flankers around an upright target), and “crowded-different”, where the orientation of the flankers differed from the target (see Fig. 2A). This resulted in 1440 trials (3 crowding conditions × 2 same/different conditions × 30 repetitions per face × 4 blocks × 2 target orientations).

### Results and Discussion

We calculated *d*′ as a measure of observers’ sensitivity to differences in eye-separation, with results shown in Fig. 2B. A two-way repeated measures ANOVA was conducted, with target orientation and crowding condition as factors. The main effect of target orientation was not significant (F[1,6] = 2.05, *P* = 0.202), indicating that there was no measurable difference in performance for upright and inverted targets. We did however find a significant main effect of crowding condition (F[2,12] = 127.35, *P* < 0.0001), as well as a significant interaction between the two factors (F[2,12] = 4.32, *P* = 0.039), which we investigated further with planned Bonferroni-corrected paired t-tests. For an upright target face, the identification of horizontal eye-shifts was strongly impaired with upright flankers and significantly less impaired with inverted (t[6] = −3.32, *P* = 0.032), as with identity judgements in Experiment 1. Crucially, performance with an inverted target was strongly impaired by inverted flankers and significantly less impaired by upright flankers (t[6] = −3.20, *P* = 0.038), unlike the pattern observed for inverted targets in Experiment 1.

We find that crowding is modulated by the orientation of the flankers for both upright and inverted target faces. This is contrary to the prediction of the holistic account of crowding that there should be no effect of flanker orientation for stimuli that are not processed holistically, such as inverted faces^19^. Furthermore, our task did not produce a significant difference between upright and inverted targets when uncrowded. If this lack of an inversion effect was taken to indicate a lack of holistic engagement, then according to Louie *et al*.^19^ we should have found no flanker modulation of crowding even with upright target faces. In fact we find the opposite: both upright and inverted target faces produced a pattern of results resembling that seen for upright faces in the identity-matching task of Experiment 1.

We argue that this symmetric pattern of target-flanker similarity emerged here due to the matched task difficulty between upright and inverted target conditions (which can be seen with uncrowded performance). This is in line with our account of crowding being selective for target-flanker similarity in all stimuli, including faces.

Alternatively, it could be argued that this general selectivity emerged in Experiment 2 because of the switch in task from identity judgements to eye judgements. Indeed it has been argued that the nature of the task can be central to the pattern of crowding^26^. As such, the selectivity for target-flanker similarity observed in this experiment could be due to the eye-judgement task primarily engaging featural (rather than holistic) processes. To rule out this interpretation, we next examined crowding using a vertical eye-judgement task that is susceptible to the inversion effect.

## Experiment 3

Although horizontal eye-shifts are minimally affected by inversion, vertical eye shifts have been shown to be disrupted in a similar fashion to identity judgements^28^. If it is the increased task difficulty resulting from inversion that obscures selectivity for target-flanker similarity, then we should find a pattern of results comparable to that observed with identity judgements (as seen in Experiment 1 and Louie *et al*.^19^). Specifically, the susceptibility of vertical-eye shifts to the inversion effect should give a modulation of crowding by flanker orientation for upright target faces, but not for inverted target faces. In contrast, if the change from identity judgements to more featural eye-shift judgements is responsible for the symmetric pattern of crowding in Experiment 2, then the same symmetric pattern should be observed with vertical eye-judgements: crowding should be modulated by the orientation of the flankers for both upright and inverted faces.

### Methods

**Figure 3:**
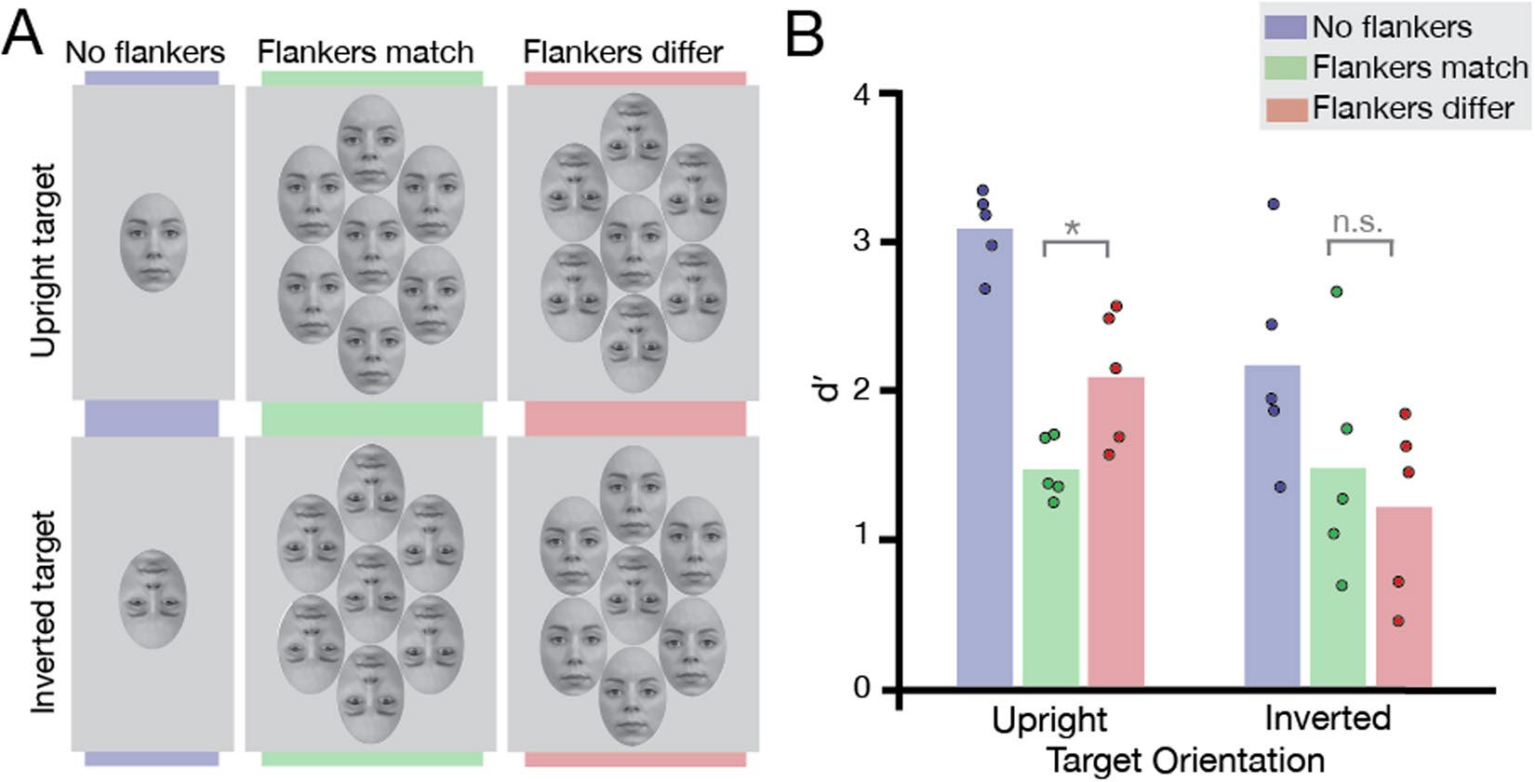
Design and results of Experiment 3 with vertical eye-judgements. (**A**) *Experimental conditions:* Two target orientations were presented: upright (top panel) and inverted (bottom panel). The crowding conditions were identical to Experiments 1 & 2. The target face could have the same vertical eye positions with the reference (“normal”, no eye-shift), or different (eyes shifted down). Face stimuli were taken from the Radboud Faces Database^32^ and edited. (**B**) *Results of Experiment 3:* Results plotted for the three crowding conditions, for upright and inverted targets (N = 5). Bars depict average *d*′ values; dots show *d*′ for each observer. Grey brackets indicate the significance level of each condition comparison (n.s. not significant, *P < 0.05).

All experimental details were identical to Experiment 2, with the following exceptions. Five observers (2 males, M_age_ = 26) took part, all with normal or corrected-to-normal vision. To construct the stimuli for this experiment we shifted the eyes and eyebrows of the reference face (RaDF-090-12^32^) by 20 pixels along the vertical axis. This face served as the target in the trials in which the target differed from the reference face (with unaltered eye positions), as well as three of the six flanker faces in crowded trials (see Fig. 3A). Stimulus sizes varied between observers, ranging from 2.3–4.0° (M = 3°) horizontally, and 3.3–5.8° (M = 4.6°) vertically. This resulted in on-screen vertical eye-shifts for each observer ranging from 0.17–0.24° (M = 0.21°). As in Experiment 2, we tested observers’ performance on two target orientations and three crowding conditions (see Fig. 3A).

### Results and Discussion

We again calculated *d*′ as a measure of sensitivity to differences in vertical eye-position (see Fig. 3B). These values were analysed using a two-way repeated measures ANOVA. Analyses revealed a main effect of crowding condition (F[2, 8] = 38.10, *P* < 0.0001). There was no main effect of target orientation (F[1, 4] = 3.40, *P* = 0.139), despite four out of five subjects performing worse with inverted target faces than upright, similar to prior work^28^. We suspect this is due to the large variability in performance for inverted targets, rather than the lack of an inversion effect *per se*, and indeed the large effect size (η^2^_partial_ = 0.46) suggests that this may be the case. Additionally, the ANOVA also revealed a significant interaction between target orientation and crowding condition (F[2, 8] = 6.44, *P* = 0.22). Planned paired t-tests with Bonferroni corrections showed that crowding followed the same pattern as in identity-matching tasks: crowding was strong for upright targets with upright flankers and significantly weaker with inverted flankers (t[4] = −3.58, *P* = 0.046). As in Experiment 1, there was no modulation of crowding by flanker orientation for inverted targets, with similarly poor performance for both upright and inverted flankers (t[4] = 0.88, *P* = 0.86).

We thus replicate the “holistic similarity” pattern of crowding with an eye-judgement task, as previously observed for identity judgements in both the study by Louie *et al*.^19^ and in our replication in Experiment 1. As such, eye-judgement tasks can also produce the pattern of results previously attributed to holistic crowding processes. Consequently, a switch in task^26^, from identity- to eye-judgements cannot explain the differing pattern of selectivity that arose in Experiment 2. We argue that the common factor in these experiments is the role of task difficulty – crowding is modulated by the orientation of the flankers when uncrowded target identification is easy (for upright target faces in all tasks and for horizontal-eye judgements with inverted targets), and is obscured when the task is difficult (for identity and vertical-eye judgements with inverted targets). Were this the case, it should be possible to obscure this modulation of crowding even for an upright target face, simply by increasing task difficulty. In other words, with increased difficulty in a task where crowding shows selectivity for target-flanker similarity, it should be possible to “turn off” the modulatory effects of the flankers. In Experiment 4 we test this by increasing the difficulty of horizontal eye-judgements, a manipulation that also allows us to further explore the role of holistic processes in eye-judgement tasks.

## Experiment 4

**Figure 4:**
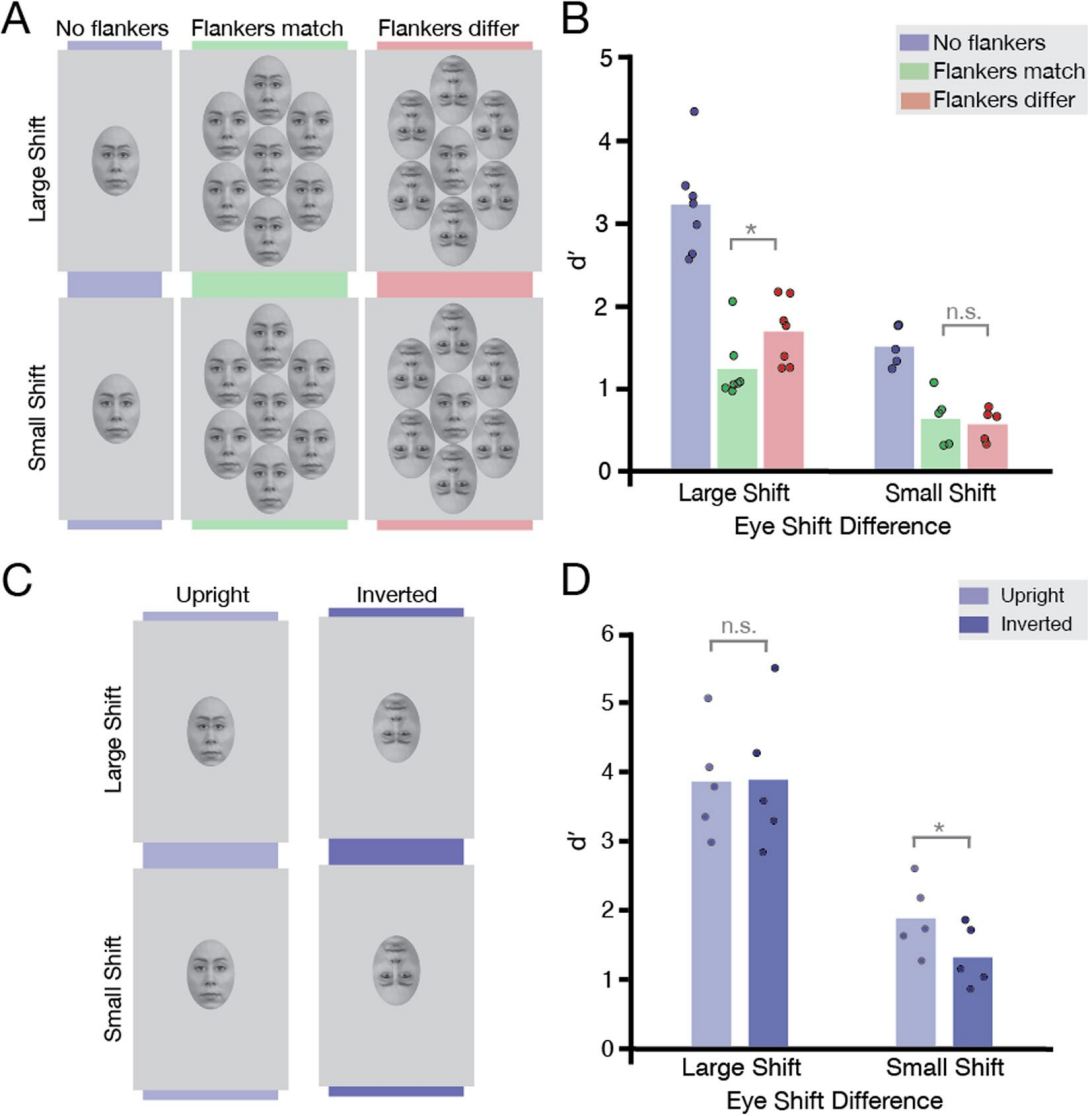
Design and results of Experiments 4a and 4b, designed to test the role of task difficulty. (**A**) *Experimental conditions of Experiment 4a:* Only upright targets were used, tested in the same crowding conditions as before: either in isolation or surrounded by either upright or inverted flankers. The top panel shows the large eye-shifts used in Experiment 2; the bottom panel shows the small eye-shifts used in Experiment 4a. The horizontal eye positions of the target were either the same (“normal”, no eye-shift) or different (inward eye shift) to the reference. Face stimuli were taken from the Radboud Faces Database^32^ and edited. (**B**) *Results of Experiment 4a:* Results plotted for upright targets in the three crowding conditions, replotted with large eye-shifts from Experiment 2 (N = 7) and small shifts from the current experiment (N = 5). Bars indicate average *d*′; dots indicate *d*′ for each observer. Grey brackets indicate the significance level of each condition comparison (n.s. not significant, *P < 0.05). (**C**) *Experimental conditions of Experiment 4b:* Targets were always presented in isolation, either upright or inverted. Upper panels show the large horizontal eye-shifts; lower panels show the small ones. (**D**) *Results of Experiment 4b:* Results plotted for upright and inverted targets for the two eye-shift conditions (N = 5). Bars indicate average *d*′; dots indicate *d*′ for each observer. Grey brackets indicate the significance level of each condition comparison (n.s. not significant, *P < 0.05).

Here, our primary aim was to investigate the role of task difficulty on judgements of horizontal eye position within upright target faces. Varying the amount of horizontal displacement allows a straightforward manipulation of task difficulty, without changing parameters such as eccentricity or target-flanker separation that would affect the magnitude of crowding^5^. If task difficulty can obscure the selectivity of crowding for target-flanker similarity in inverted target faces (making both upright and inverted flankers equally disruptive to identification), then by increasing the difficulty of our horizontal eye-judgement task (by decreasing the displacement of the eyes), it should also be possible to disrupt the selectivity for target-flanker similarity in *upright* target faces. Were this possible, upright and inverted flankers should produce equivalent levels of crowding for an upright target. Alternatively, if task difficulty does not modulate the effect of crowding on faces, then the recognition of upright target faces should be more disrupted by upright than inverted flankers in all cases where uncrowded performance is above chance. We examine this possibility in Experiment 4a (Fig. 4A).

Previous studies^37^ have in fact demonstrated that when differences in facial features are more subtle, holistic processes are more strongly engaged. As such, horizontal eye judgements with a reduced displacement in the eyes should not only become more difficult, but may also engage holistic processes to a *greater* degree than the larger displacements in Experiment 2. If crowding is modulated by holistic similarity, this increased holistic engagement should increase (or at least maintain) the difference in crowding between upright and inverted flanker conditions. In order to assess this possibility, in Experiment 4b (Fig. 4C) we compared uncrowded performance with upright and inverted targets for both small and large eye displacements to seek the presence of an inversion effect (the classic indicator of holistic engagement^20–24^). If horizontal eye judgements can engage holistic processes when feature differences are subtle, we should find an inversion effect with small eye displacements. If crowding is sensitive to holistic similarity, the presence of an inversion effect with these small displacements should coincide with a difference between the upright and inverted flanker conditions in Experiment 4a. In contrast, an inversion effect for small displacements in Experiment 4b in the absence of any difference between flanker conditions in Experiment 4a would provide further evidence that crowding does not rely on holistic similarity.

### Methods

#### Experiment 4a

Methods were identical to those of Experiment 2, with the following exceptions. Five observers (2 females, M_age_ = 27.4 years) who participated in Experiment 2 took part. To increase task difficulty, the displacement of the horizontal eye position was reduced compared to Experiment 2: the eyes and eyebrows of face RaDF-090-12^32^ were shifted inwards by 10 pixels (half the displacement of Experiment 2). Stimulus sizes varied between 2.1–3.6° (M = 3.1°) horizontally and 3.0–5.2° (M = 4.5°) vertically, leading to on-screen horizontal eye displacements of 0.08–0.13° (M = 0.11°). As in Experiment 2, observers were shown peripheral target faces at 12° eccentricity that were either unaltered or shifted inwards, and were required to judge whether the target face was the same or different from the reference with regards to horizontal eye position. As the target face was always upright, this resulted in three crowding conditions: “uncrowded”, “crowded-same, and “crowded-different” and 720 trials (3 crowding conditions × 2 same-different conditions × 30 repetitions per face × 4 blocks).

#### Experiment 4b

Five observers (2 males, M_age_ = 31.5 years) took part, all with normal or corrected-to-normal vision. Stimuli were as in Experiment 4a, though only the target-alone uncrowded conditions were used. Stimulus sizes varied between 2.0–3.9° (M = 3.1°) horizontally and 2.9–5.4° (M = 4.5°) vertically. This led to on-screen horizontal eye displacements of 0.15–0.27° (M = 0.22°) for the “large shift” condition and 0.07–0.14° (M = 0.11°) for the ‘small shift’ condition. As in Experiments 2 and 4a, observers judged whether the target, presented at 12° eccentricity, was the same or different from the reference face in terms of eye position. The target was always presented in isolation (“uncrowded”) and was either upright or inverted, resulting in 480 trials per eye shift condition (2 same-different conditions × 30 repetitions per face × 4 blocks).

### Results and Discussion

As in Experiment 2, *d*′ was calculated as a measure of observers’ sensitivity to differences in eye separation. Results for Experiment 4a are shown in Fig. 4B. It is clear that the smaller eye shifts were harder to detect even when the target was uncrowded (plotted in Fig. 4B against data with large displacements from Experiment 2). Nonetheless, crowding still had a significant effect on identifying differences in horizontal eye displacement: Bonferroni corrected t-tests showed that performance with uncrowded targets was better than in conditions where the target was surrounded by flankers of the same (t[4] = 8.23, *P* = 0.003) or different orientation (t[4] = 6.04, *P* = 0.012). Importantly however, when crowded, there was no significant difference between observers’ ability to detect small eye displacements with upright vs. inverted flankers (t[4] = 0.5, *P* > 0.99). The equivalent difficulty of these two conditions resembles the pattern of results for inverted targets in identity-matching tasks (Experiment 1).

These results provide direct evidence that an increase in task difficulty can obscure the selectivity of crowding for target-flanker similarity, even for upright target faces. Here we produce this effect using the same horizontal eye-judgements previously shown in Experiment 2 to produce a clear selectivity for target-flanker similarity. We argue that as performance drops for uncrowded targets, the range over which performance can vary in crowded conditions becomes restricted, thereby obscuring any differences between the upright and inverted flanker conditions. We explore this mechanism further in the General Discussion.

One criticism is that horizontal eye-judgement tasks may not engage holistic processes, and that the pattern observed in Experiments 2 and 4a is a result of this. The results of Experiment 4b, plotted in Fig. 4D, contradict this view. As in Experiment 2, the identification of large eye-shifts is equally difficult in upright and inverted faces, with no significant difference in performance between upright and inverted target conditions (t[4] = −0.14, *P* = 0.849, Bonferroni corrected). However, with small eye-shifts, we find a significant difference in performance between upright and inverted target conditions (t[4] = 3.76, *P* = 0.039, Bonferroni corrected), with small eye shifts being more difficult to identify in inverted faces. This inversion effect indicates that holistic processes are engaged when horizontal eye judgements are sufficiently subtle, in line with previous findings demonstrating a greater engagement of holistic processes when the discriminability of facial features is decreased^37^.

We note that overall performance in the small eye-shift condition is lower than that reported in previous studies using eye shifts of similar magnitude, for stimuli presented foveally^28,38^. This is likely due to the peripheral presentation of our stimuli, with resulting decreases in visibility via reduced acuity and contrast sensitivity^2^, and a probable increase in within-face crowding^35^. Our inversion effect is similarly smaller than that found in these prior studies, though it is consistent with the range of values found more generally^39,40^. Nonetheless, the inversion effect that we observe, along with those found in prior studies^28,38^, suggests the potential for holistic processes to be engaged in judgements of horizontal eye separation.

These findings provide further evidence that the differing pattern of results in Experiments 1 and 2 did not arise simply due to our use of an identity task in the former and an eye-judgement task in the latter. In Experiment 4b, with small eye-shifts, our horizontal eye-judgement task shows a significant inversion effect, the typical marker of holistic engagement^20–24^. If the “holistic similarity” criterion of crowding were to operate in a similar fashion, then we should have observed a *stronger* modulation of crowding in the small eye-shift condition of Experiment 4a – that is, the difference between upright and inverted flanker conditions should have increased (or at the very least, to have remained present). In contrast, we find no modulation of crowding by the orientation of the flankers on an upright face. This finding cannot be attributed to the task engaging primarily local processes, given the presence of a significant inversion effect for the same task in Experiment 4b. Rather, our findings together demonstrate that the common factor that obscures the release from crowding is task difficulty, rather than any propensity for crowding to operate holistically – with high difficulty there is a lack of flanker modulation for crowding (here for upright faces), which can be revealed when difficulty is lowered (as in Experiment 2, with inverted faces).

## Experiment 5

If task difficulty can account for the asymmetry in the crowding of faces (seen in Experiments 1 and 3), and holistic similarity does not determine the strength of crowding, what is driving the difference in crowding with upright and inverted flankers when it occurs? It is important to consider here that inversion not only reduces the capacity for the holistic processing of faces, but also alters the features within faces. Relative to an upright face, inversion alters the orientation of each feature (i.e. the nose rotates to be upside-down), as well as the spatial order of these features (the eyes above nose above mouth pattern is reversed)^28,41^. More specifically, the spatial order of the features results in a predominantly top-heavy pattern in upright faces that becomes predominantly bottom-heavy (in a retinotopic sense) when they are inverted^42,43^. Similar differences in feature positions have previously been shown to modulate crowding within letter-like stimuli – an upright T will be crowded less by inverted T flankers than by other configurations^44^, for instance. In our final experiment, we manipulated both the orientation of facial features and their spatial order independently in order to assess the contribution of these dimensions to the selectivity of face crowding for target-flanker similarity.

### Methods

Μethods were similar to Experiment 2, with the following exceptions. Five observers (4 females, M_age_ = 25) took part, all with normal or corrected-to-normal vision. The target and reference faces were always the face RaDF-090-12^32^, either with no eye shift or with the eyes and eyebrows displaced by 20 pixels inwards (as in Experiment 2). Stimulus sizes for each observer ranged between 2–4° (M = 3.2°) horizontally and 2.9–5.8° (M = 4.7°) vertically. This resulted in horizontal eye displacements between 0.15–0.36° (M = 0.26°) for stimuli presented on-screen.

**Figure 5:**
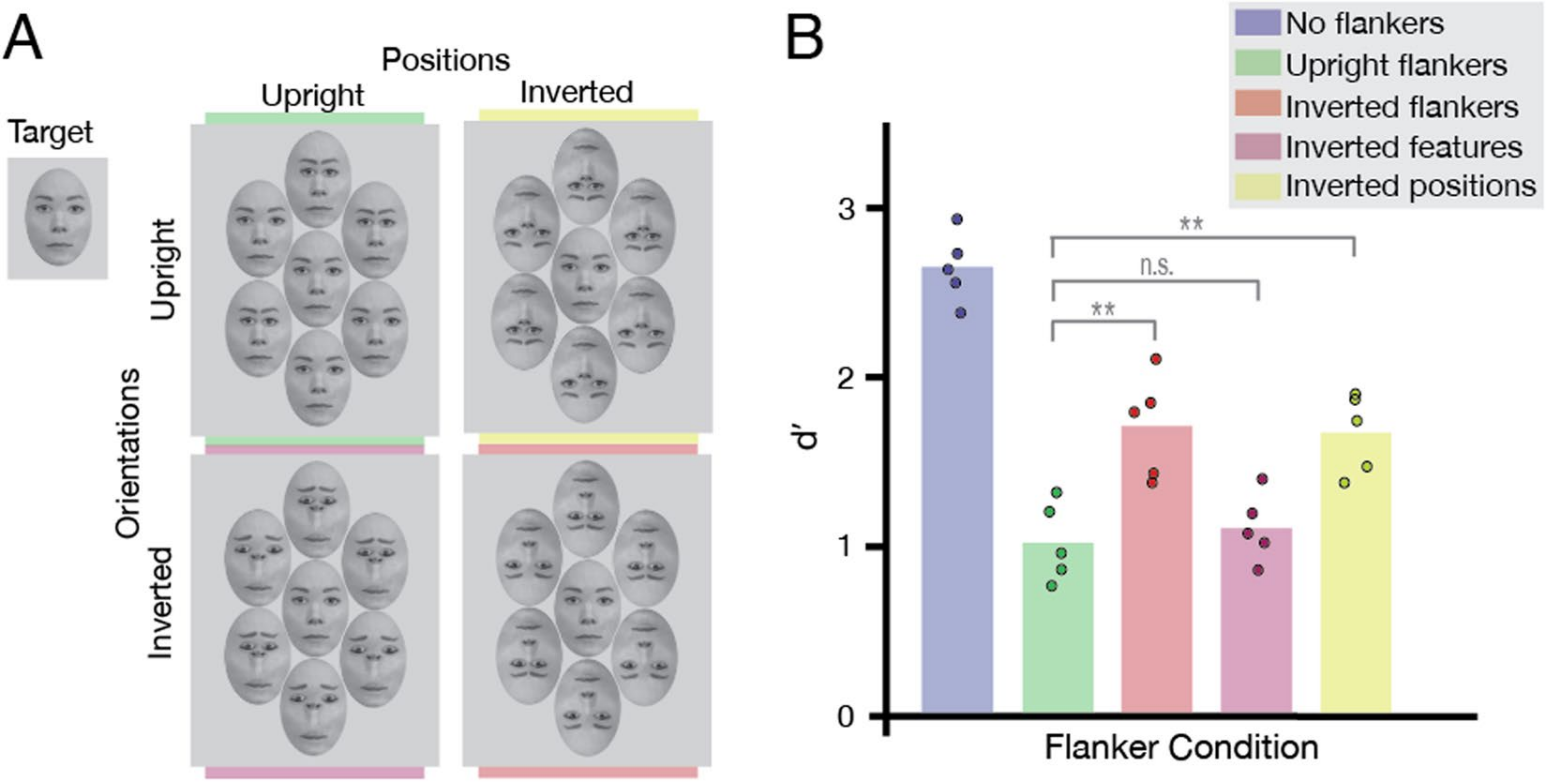
Design and results of Experiment 5, designed to test the selectivity of face crowding. (**A**) *Experimental conditions:* Only upright target faces were presented, with five flanker conditions: no flankers, flankers with upright positions and features (top left, upright flankers), flankers with inverted positions and features (bottom right, inverted flankers), flankers with inverted features but upright positions (bottom left, “inverted features”), and flankers with upright features but inverted positions (top right, “inverted positions”). Face stimuli were taken from the Radboud Faces Database^32^ and edited. (**B**) *Results of Experiment 5: *Results plotted for the five flanker conditions for upright targets (N = 5). Bars depict average *d*′; dots indicate *d*′ for each observer. Grey brackets indicate the significance level of each condition comparison (n.s. not significant, *P < 0.05, **P < 0.01).

We used the reference face with and without the horizontal displacements to manipulate the features and first-order relational properties^41^ of the flankers independently. To examine the effect of feature orientations, we rotated the facial features of the faces (to match an inverted face), whilst keeping their positions in the same spatial order as the upright target (Fig. 5A, bottom panel). To examine the effect of the spatial order of facial features, we shifted their position in the faces (to match inverted faces) without rotating them (to match the upright target; Fig. 5A, top right panel). These two new “Thatcherised”^45^ faces, as well as the upright and inverted faces used in Experiment 2, served as the four different flanker conditions in this experiment.

In this experiment, the target face was always upright, with five crowding conditions, including “uncrowded”. These conditions with their relevant face flankers can be seen in Fig. 5A. In the first two crowded conditions, flankers were identical to the previous experiments: either upright flankers surrounding an upright target, or inverted flankers surrounding an upright target. In the third “inverted features” condition, the feature orientations were altered (i.e. rotated to be consistent with an inverted face) but their positions were held constant (consistent with the spatial order of features in an upright face). For the final “inverted positions” condition, the facial feature orientations were held constant (to be consistent with an upright face) while the positions were rotated (to match the spatial order of an inverted face). This resulted in 1200 trials (5 crowding conditions × 2 same/different conditions × 15 repetitions per face × 2 target faces × 4 blocks).

### Results and Discussion

Observers’ performance in detecting horizontal eye-shifts was measured in the five flanker conditions (Fig. 5A), with *d*′ again computed as an indicator of sensitivity to differences in eye-separation (Fig. 5B). A one-way repeated measures ANOVA revealed a significant main effect of crowding condition (F[4,16] = 99.85, *P* < 0.0001). Planned t-tests with Bonferroni corrections revealed that as in the other experiments, there was strong crowding when an upright-face target was surrounded by upright flankers and significantly weaker crowding with inverted flankers (t[4] = −6.001, *P* = 0.012). Importantly, when flankers had rotated features but kept the spatial order of an upright face (“Inverted features” in Fig. 5B), crowding remained strong and performance did not differ from that with upright flankers (t[4] = −1.566, *P* = 0.579). Crowding was however weakened (leading to improved performance) when flankers had upright features re-arranged to match the spatial order of an inverted face (“Inverted positions” in Fig. 5B) – performance in this condition was significantly better than performance with upright flankers (t[4] = −8.54, *P* = 0.003).

Our findings thus demonstrate that it is the differences in the spatial order of facial features between target and flanker elements that drives the selectivity of upright face crowding. Although this spatial order is likely a key aspect for the engagement of holistic processing^21–23^, the results of Experiments 2, 4a, and 4b indicate that these differences in the spatial order between target and flanker elements can modulate crowding regardless of the degree of holistic engagement by the task. If this task were to exclusively drive featural (rather than holistic) crowding^35^, we should have observed a release from crowding with the inversion of facial features in the flankers. However, this condition produced an equivalent degree of crowding to upright flanker faces. The differences in crowding that we observe with inverted flanker faces and flankers with inverted feature positions (but upright feature orientations) suggest that it is the spatial order of faces that modulates crowding between faces, not the extent of holistic engagement.

Because upright faces are top-heavy stimuli, inversion changes the distribution of this content across the image, making inverted faces bottom-heavy^42,43^. When the spatial order of flanker features is changed by inverting their positions (whilst keeping their orientation constant), the flanker faces become similarly bottom heavy, again leading to a release from crowding with an upright (top-heavy) face. In contrast, changing the orientation of the features whilst maintaining upright spatial order in the flankers maintains the top-heavy content, and crowding remains strong. We have further quantified these differences in image content (see Supplementary Materials and Supplementary Fig. 1), and show that spatial order variations can well account for our results in this experiment. We further demonstrate that changes in the top-heavy nature of faces can also account for findings regarding “holistic crowding” obtained with Mooney faces^25^ (Supplementary Fig. 2). The lack of orientation selectivity observed by Louie *et al*.^19^ for houses may conversely reflect the more vertically balanced nature of the image content in this stimulus set – these cropped sections of house images are unlikely to produce a consistent difference in image content between upright and inverted elements. In sum, we argue that it is differences in the spatial order within target and flanker stimuli that modulates crowding, regardless of their propensity for holistic processing.

## General Discussion

We have shown that crowding is driven by a general selectivity for target-flanker similarity, even in complex stimuli such as faces. In Experiment 1, we replicated the asymmetric pattern of crowding observed in Louie *et al*.^19^ – for these identity judgements, crowding was strong for an upright target face surrounded by upright flankers, and weak with inverted flankers. In contrast, with an inverted target face, crowding was strong with both upright and inverted flankers. This asymmetry was previously argued to reflect the selectivity of an additional stage of crowding sensitive to holistic similarity in faces^7,19,25^, independent from the crowding of inverted faces and simple objects.

On the contrary, in Experiment 2, we examined judgements of horizontal eye separation, known to show minimal inversion effects^28^, and found that crowding was strong when the orientation of the flankers matched the target and weak when it differed for *both* upright and inverted target faces. The symmetric selectivity that we observe with this task is opposed to the predictions of the “holistic similarity” account, whereby crowding should be modulated by the orientation of the flankers only for an upright holistically-processed target face. Our observation of this pattern with both upright and inverted target faces is therefore inconsistent with a distinct process for the crowding of upright faces. Rather, our results follow the more general pattern seen for a range of fundamental visual features, including orientation^6,9,10^, colour^11–13^, position^18^, motion^13^, and spatial frequency^17^, suggesting the operation of a general mechanism.

Why then does crowding show an asymmetric pattern of selectivity in some tasks but not others? Experiment 4a shows that when horizontal eye shifts become more difficult to detect, crowding for an upright target face is as strong with inverted flankers as with upright flankers, a pattern that ordinarily occurs for inverted faces in tasks such as identity matching^19^ (replicated in Experiment 1) and vertical eye-judgements (Experiment 3). Experiment 4b further demonstrates that these small horizontal eye shifts actually engage holistic processes to a greater extent than do larger shifts (consistent with prior work^37^). If crowding were operating holistically, one would therefore expect greater modulation of crowding by the orientation of the flankers in this case, rather than the lack of modulation that we observe. Rather, it is the increase in task difficulty, and not a lack of holistic processing, that can obscure the symmetric selectivity of crowding for target-flanker similarity, even for upright faces. The lack of apparent selectivity in these cases of high task difficulty can be attributed to a restriction in the range over which observers’ performance varies. By decreasing performance for uncrowded targets, task difficulty restricts the upper bound for the release from crowding. In the case of an inverted target face, the difficulty in making identity judgements obscures these modulations because the decline in uncrowded performance leaves little room for improvement.

In fact, similar effects of task difficulty can also be observed in previous studies using judgements of identity and gender that clearly engage holistic processes. In Experiment 1 of Louie *et al*.^19^, increasing levels of random dot noise were applied to the upright target face, making identity judgements gradually more difficult. With this increase in difficulty, crowding became equally strong with upright and inverted flankers at the fourth level of noise used in their study (their Fig. 1D). This effect was not due to a floor effect with uncrowded targets, as uncrowded performance remains above chance levels, meaning that the addition of the flankers could still make identity judgements harder. Nonetheless, upright target faces in this case were no longer modulated by the orientation of the flankers. A similar effect can be observed for gender judgements used to examine crowding with Mooney faces^25^. Farzin *et al*.^25^ found that the recognition of uncrowded upright Mooney faces became more difficult with increasing eccentricity (their Figure 8C). Although an effect of target-flanker differences in face orientation was observed at eccentricities of 3° and 6°, at 10° these judgements became sufficiently difficult that the modulation disappeared. Once again, uncrowded judgements remain above chance, and yet the pattern of selectivity for target-flanker similarity disappears.

We have explored the effect of task difficulty further with a population model that is similar in principle to recent models of the effect of crowding on orientation^46,47^. By simulating a population response to interocular eye distance, consistent with psychophysical^48,49^ and physiological findings^50,51^, we can similarly model the crowding of faces as a pooling process that combines responses to the target and flanker elements. With this model, we demonstrate that an increase in task difficulty (produced by decreasing the interocular separation for horizontal eye judgements, as in Experiment 4) can decrease the separability of target and flanker signals (see Supplementary Fig. 3). Although an increase in target-flanker differences would ordinarily allow the target response to be clearly separated from flanker responses, an increase in task difficulty must be accompanied by a greater reduction in crowding to give the same separation in the population response. Similar complications would likely arise for judgements of identity with inverted faces. More generally, these effects of task difficulty may also explain “supercrowding” effects, where the masking of a target can impair its identification beyond the typical range of interference zones for crowding^52^.

Our final experiment demonstrates that the crowding of faces is driven by a selectivity for the spatial order of facial features. Crowding is strong when the spatial order of facial features (i.e. their first-order relations) are matched between target and flankers, and weak when they differ, regardless of individual feature orientations. This effect of spatial structure is broadly consistent with findings^53^ that schematic faces and electrical-plug flankers crowd photographic faces more than Chinese characters (although these stimuli did not produce an effect of flanker orientation, likely due to the differences in texture, contrast, and spatial frequency content between target and flanker elements). The spatial order of facial features is a key aspect of holistic processes in face perception^21–23^, perhaps driven by the top-heavy^42,43^ vertical ordering of horizontal structure^54–56^. However, the results of Experiment 2 demonstrate that crowding is selective for target-flanker differences in this spatial order even with inverted target faces, irrespective of their capacity for holistic processing. With the photographic face stimuli used in our study, this selectivity could be driven by image-based differences in the vertical configuration of horizontally oriented image structure (Figs S1 and S2). Although these differences in spatial structure clearly drive higher-level areas^57^, spatial variations of this nature could be processed in cortical areas as early as V1^58^, V2^59,60^ or V4^61^, all of which have been implicated in the general operation of crowding^62–67^. Spatial order has also been shown as relevant for crowding more generally, through its modulation by contours^68,69^, spatial regularity^70–72^, and the structure of letter-like stimuli^44^. There is therefore is no need to invoke multiple processes of crowding to explain the selectivity for target-flanker similarity in upright faces – changes in the spatial order of features within upright or inverted faces can modulate the strength of crowding via these more general processes.

Our argument that face crowding can be explained by a combination of selectivity for spatial order and task difficulty could also explain similar “holistic” findings for crowding. In particular, crowding for upright Mooney faces^73^ has been shown to be strong with upright Mooney flankers, and weak with inverted flankers^25^. As with photographic faces (Fig. S1), our image analyses show that upright Mooney faces are top-heavy stimuli, with the majority of their contours concentrated at the top of the face image, whereas inverted Mooney faces are bottom-heavy stimuli (Fig. S2). Furthermore, although the visibility of individual features is degraded in Mooney faces, prior work shows that their contours remain susceptible to within-face crowding in much the same fashion as photographic face images (see Experiment 3 of Farzin *et al*.^25^). This identical susceptibility to inter-feature crowding suggests that similar processes can account for crowding in both stimulus types. A shift from top- to bottom-heavy spatial order in the Mooney flankers (relative to an upright target) would therefore be expected to reduce crowding, similar to the spatial-order effects seen with other stimuli^44,68–71,72^. As such, target-flanker differences in spatial order, rather than their propensity for holistic processing, could also drive the crowding of Mooney faces. The shift in the spatial order of image content could also drive crowding for other stimuli that have canonical upright configurations like faces, such as Chinese characters^74^ and biological-motion arrays^75^.

Altogether, our results demonstrate that when task difficulty is matched between upright and inverted target conditions, crowding for faces is selective for differences in the spatial order of facial features. We argue that this reflects a general selectivity for target-flanker similarity that determines the strength of interactions between stimuli ranging from faces^19^ to facial features (“within–face” crowding)^35^ and simple objects^8,16,18^. In other words, there is no need for an independent high-level crowding process that is selective for holistic similarity^19,25^ – crowding is likely to operate in the same way for all stimuli. Note that this does not contradict evidence for other higher-level effects in crowding, including the Gestalt grouping processes that have been shown to modulate crowding in multi-element arrays^70,76,77^. Additionally, our account is not necessarily inconsistent with the involvement of multiple visual processing stages^7,26^ or multiple visual brain areas^62,63,66,78^ in crowding. Given the wide range of stimuli susceptible to crowding (from colour patches and moving Gabors to faces), distinct cortical regions or populations of neurons may be involved in the computations that produce the disruption within each feature domain. In this sense, we cannot entirely exclude the possibility that the crowding of faces occurs in a distinct stage – we simply demonstrate here that the behavioural evidence for this possibility (based on differences in the selectivity of crowding) does not hold up to scrutiny. Importantly, even if separate processing stages are required for crowding, our results suggest that each should operate in the same fashion – with a common selectivity for the visual similarity between target and flanker objects.

## Electronic supplementary material

Supplementary information
