# Supplementary material for: Crowding for faces is determined by visual (not holistic) similarity: Evidence from judgements of eye position
Source: Sci Rep. 2018 Aug 22;8:12556. doi: 10.1038/s41598-018-30900-0 (PMC6105622; doi:10.1038/s41598-018-30900-0)
Supplement: Supplementary file 1 — Supplementary information [file 41598_2018_30900_MOESM1_ESM.pdf]

Supplementary information for:

Crowding for faces is determined by visual (not holistic) similarity: Evidence from judgements of eye position

Alexandra V. Kalpadakis-Smith[1], Valerie Goffaux[2,3,4], & John A. Greenwood[1]

[1] *Experimental Psychology, University College London, London, United Kingdom*
[2] *Research Institute for Psychological Science, Université Catholique de Louvain, Louvain-la-Neuve, Belgium*
[3] *Institute of Neuroscience, Université Catholique de Louvain, Louvain-la-Neuve, Belgium*
[4] *Department of Cognitive Neuroscience, Maastricht University, Maastricht, The Netherlands*

Quantifying target-flanker similarity for our stimuli

In this section we consider the specific visual dimensions that underlie the selectivity of face crowding for target-flanker similarity. As well as reducing the propensity for holistic processing[1-4], the inversion of a face alters both the orientation of the facial features and the spatial order of these features (i.e. the eyes above nose above mouth pattern is reversed)[5]. Differences between the target and flanker faces in either of these properties could be driving the variations in crowding that we observe. In Experiment 5, we independently assessed the contribution of the orientation and order of facial features in crowding by introducing two additional types of "Thatcherised"[6] flanker faces. First, to examine the role of feature orientation, we constructed flankers with rotated facial features (to match an inverted face) where the position of features was maintained in the same first-order pattern as an upright face ("inverted features"). Second, to examine the role of the spatial order of the features, we shifted the position of facial features (to match inverted faces) without rotating them to maintain similarity with an upright face ("inverted positions").

In order to consider the image-based differences introduced by these different flanker stimuli, we computed the Fourier energy for each flanker type in a range of spatial frequency and orientation bands. Analyses were conducted on four faces: the upright target face, an inverted version of the face (used in our experiments as both target and flankers), and the two Thatcherised flanker faces with either inverted features or inverted positions. Images were padded with grey values to extend image dimensions to 1024×1024 pixels. Each image was then fast Fourier transformed before the amplitude spectrum was multiplied with wrapped Gaussian filters with a standard deviation of 20°, centred on orientations between 0° (horizontal) and ±90° (vertical) in 15° increments. Twenty peak spatial frequencies were tested in linear steps between 1-30 cycles per image and a bandwidth of one octave. After the inverse Fourier transform, the RMS contrast of all resulting images was adjusted to match the average RMS contrast of the original image set. Images were then cropped to retain only the central 512×512 pixels to avoid edge artefacts. Example images computed from this stage are shown in Supplementary Figure 1A.

When the energy within the image is summed across all spatial frequencies, it is apparent that each image contained considerably more energy within horizontal bands than within vertical or oblique bands (Supplementary Figure 1B), consistent with previous reports[7,8]. The overall image content of these faces is therefore highly consistent (particularly since the upright and inverted faces are simply vertically flipped versions of each other, as are the two Thatcherised faces).

To characterise the differences in the target and flanker faces that could drive the crowding of an upright target face (as observed in Experiment 5), we next subtracted each filtered face from the upright filtered face, separately within each spatial frequency and orientation band (on a pixel-by-pixel basis), and again summed the energy across the image. The resulting image differences were then squared and summed to compute the total energy difference between the faces. The average difference (across spatial frequency) within each orientation band is plotted in Supplementary Figure 1C. Firstly, the subtraction of an upright face from itself necessarily produces a zero difference in energy (data not shown). If we instead subtract an inverted face from the upright target (red line in Figure S1C), there are image differences at all orientations, though this is clearly more so in the horizontal bands than in the vertical or oblique ranges. The difference between an upright face and the Thatcherised face with inverted positions follows a nearly identical pattern, with extremely similar values (yellow line in Figure S1C). In contrast, the differences between an upright face and the Thatcherised face with inverted features (in the same positions as the upright face) are considerably reduced at all orientations (purple line in Figure S1C).

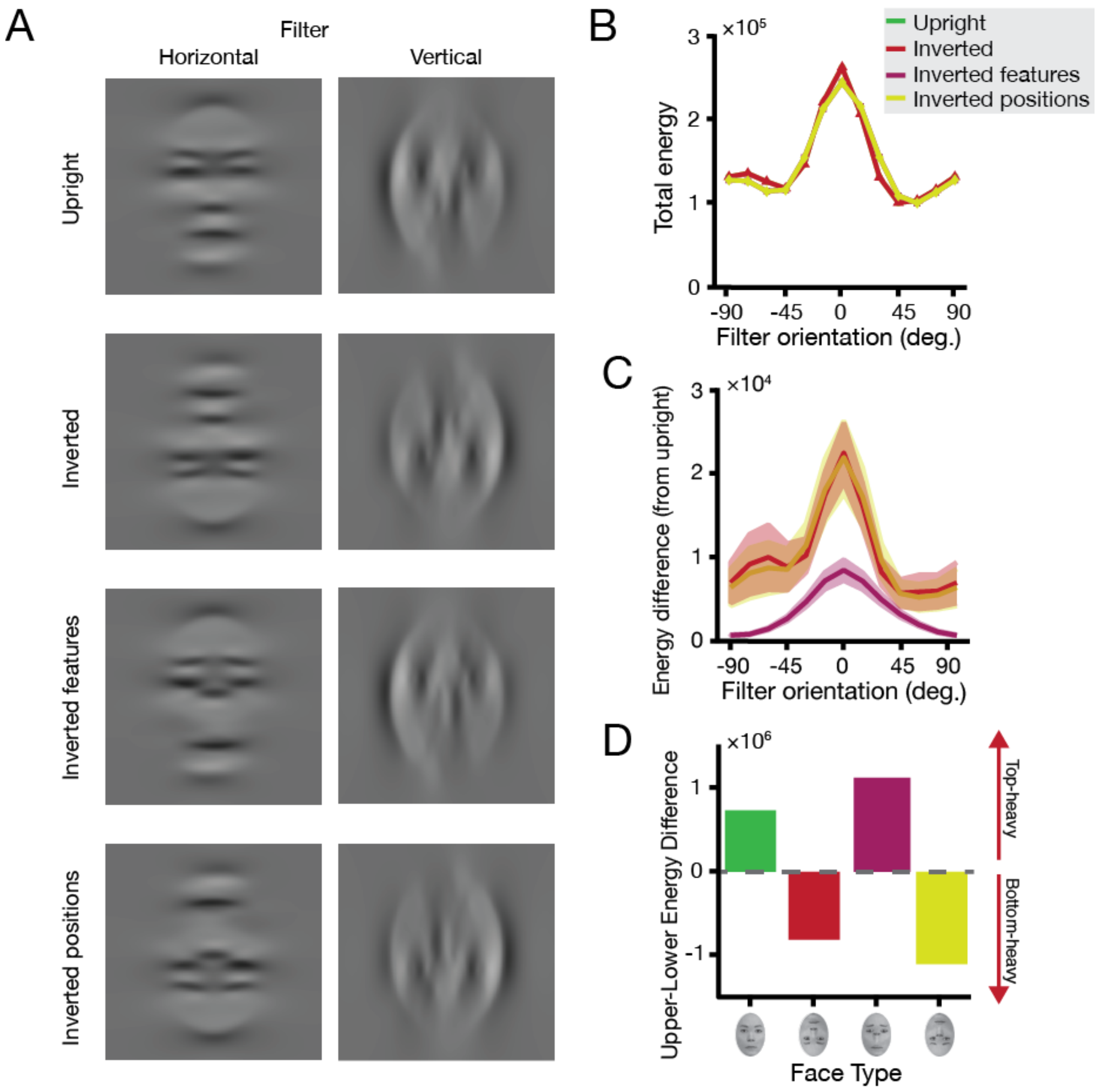

Figure S1: Image analyses for face stimuli

A) Example face stimuli, taken from the Radboud Faces Database[9] and edited as described in the Methods section of Experiment 5, after convolution with log-Gaussian filters in the Fourier domain. All images are shown after convolution with a peak spatial frequency of 5 cycles per image. The first column shows the following horizontally filtered faces (from top to bottom): an upright face, an inverted face, a Thatcherised face with inverted features, and a Thatcherised face with inverted positions. The second column shows the same faces but vertically filtered.
(B) The total Fourier energy in each image, summed across spatial frequencies within each orientation band (where 0° = horizontal and 90° = vertical). Note that because upright and inverted faces are simply flipped versions of the same stimulus, their values overlap completely. The same is true for the two Thatcherised faces.
(C) Differences in Fourier energy between each face type and an upright face, plotted as a function of the orientation band. Lines show the average and shaded regions show the 95% confidence interval across spatial frequencies. Note that the difference between upright faces lies at zero.
(D) Differences in summed contrast energy between the upper and lower halves of each image used in Experiment 5. Positive values show 'top-heavy' and negative values show 'bottom-heavy' configurations.

In part, these differences in image structure arise because faces are 'top-heavy' stimuli with greater contrast variations in the upper half of the image (primarily due to the eyes and eyebrows) than the lower half[10,11]. We can quantify this in our stimuli by summing the squared contrast energy at all orientations and spatial frequencies within the lower-half of the image and subtracting this from the sum of squared contrast energy in the upper-half of the image. These values are shown in Figure S1D for the four stimuli used in Experiment 5, where positive values indicate 'top-heavy' stimuli and negative values show 'bottom-heavy' stimuli. Here it is apparent that an upright face is top-heavy, as is the 'inverted features' stimulus with features rotated in place. By contrast, both the inversion of the face image and the inversion of feature positions (with feature orientations kept constant) produces bottom-heavy stimuli.

The image-level differences in Supplementary Figures 1C and 1D show a strong similarity with the results from Experiment 5 (shown in Figure 5). The current analyses show that the inversion of a face produces strong changes in the spatial order of the image, particularly for horizontally oriented structure, with a shift from a 'top-heavy' to a 'bottom-heavy' configuration. In Experiment 5, this difference was associated with a reduction in crowding with inverted-face flankers, relative to the crowding produced by upright flankers. Similar changes in image content are produced by re-arranging the positions of the upright features to match an inverted face – accordingly, in Experiment 5 we observe a clear reduction in crowding with these flankers. There was far less change in image content when the features were rotated in place, which indeed produced no change in crowding (relative to that produced by upright flankers).

The differences between upright and inverted faces in the distribution of image content have previously been used to examine holistic processes in face recognition, showing that horizontal content is a more effective driver of the behavioural signatures of configural processing[7,12,13], and that top-heavy stimulus configurations drive infants' looking preferences[10,11]. However, with crowding it is important to note the relations between target and flanker stimuli along these dimensions. Here we show that this image content differs markedly between upright

and inverted faces, as well as with Thatcherised faces with large changes in the spatial ordering of features. These differences in image content could determine the strength of crowding in face stimuli in a similar fashion to the changes produced by other differences in dimensions such as colour[14-17], contrast polarity[18], and orientation[19-22]. For instance, differences in feature positions of this kind have previously been shown to modulate crowding within letter-like stimuli – an upright T will be crowded less by inverted T flankers than by other configurations[23]. We consider these mechanisms further in the General Discussion.

Quantifying target-flanker similarity with Mooney faces

The results of Experiment 5, combined with the above analyses of the orientation energy within face stimuli, suggest that the crowding of face stimuli is driven by target-flanker similarity in the top-heavy vertical configuration of horizontally oriented image structure, similar to the ‘bar codes’ argued to influence holistic processing in general[24,25]. However, evidence for an holistic stage of crowding derives not only from the recognition of photographic face stimuli[26], but also from the usage of Mooney faces[27] where local image content is much more difficult to discern[28]. As we argue in the General Discussion, although it is true that these stimuli are degraded in terms of the visibility of their local image features (e.g. it is often difficult to make out a nose from an isolated patch of the image), it is not the case that Mooney faces contain no features at all. For instance, Experiment 3 of the study by Farzin et al.[27] demonstrates that these stimuli are susceptible to self-crowding between local features in a similar way to photographic images of faces[29]. Mooney faces also contain a spatial configuration of oriented image structure that is, by and large, similar to that of regular faces[24]. Here we consider the effect of inversion on this image structure and how this may interact with crowding.

To examine the image content of Mooney faces, an identical set of analyses was performed for a set of Mooney faces as performed on our photographic face stimuli above. A set of 24 Mooney faces was obtained from the Mooney-MF database within the Psychological Image Collection at the University of Stirling[*]. 12 were male and 12 female, with several faces that match those used previously by Farzin et al.[27]. To match stimuli to those analysed above, images were reduced to the same dimensions (395×292 pixels), with an oval-shaped aperture with the same dimensions then placed around stimuli to match the image shape required for presentation as crowding stimuli (as in Farzin et al.[27]). Two example images are shown in Supplementary Figures 2A and 2B.

What is immediately apparent upon examining these Mooney images (and example images in the study of Farzin et al.[27]) is their increased variability relative to standard face stimuli. In addition to standard frontal views of the face,

[*] Available online at http://pics.stir.ac.uk/

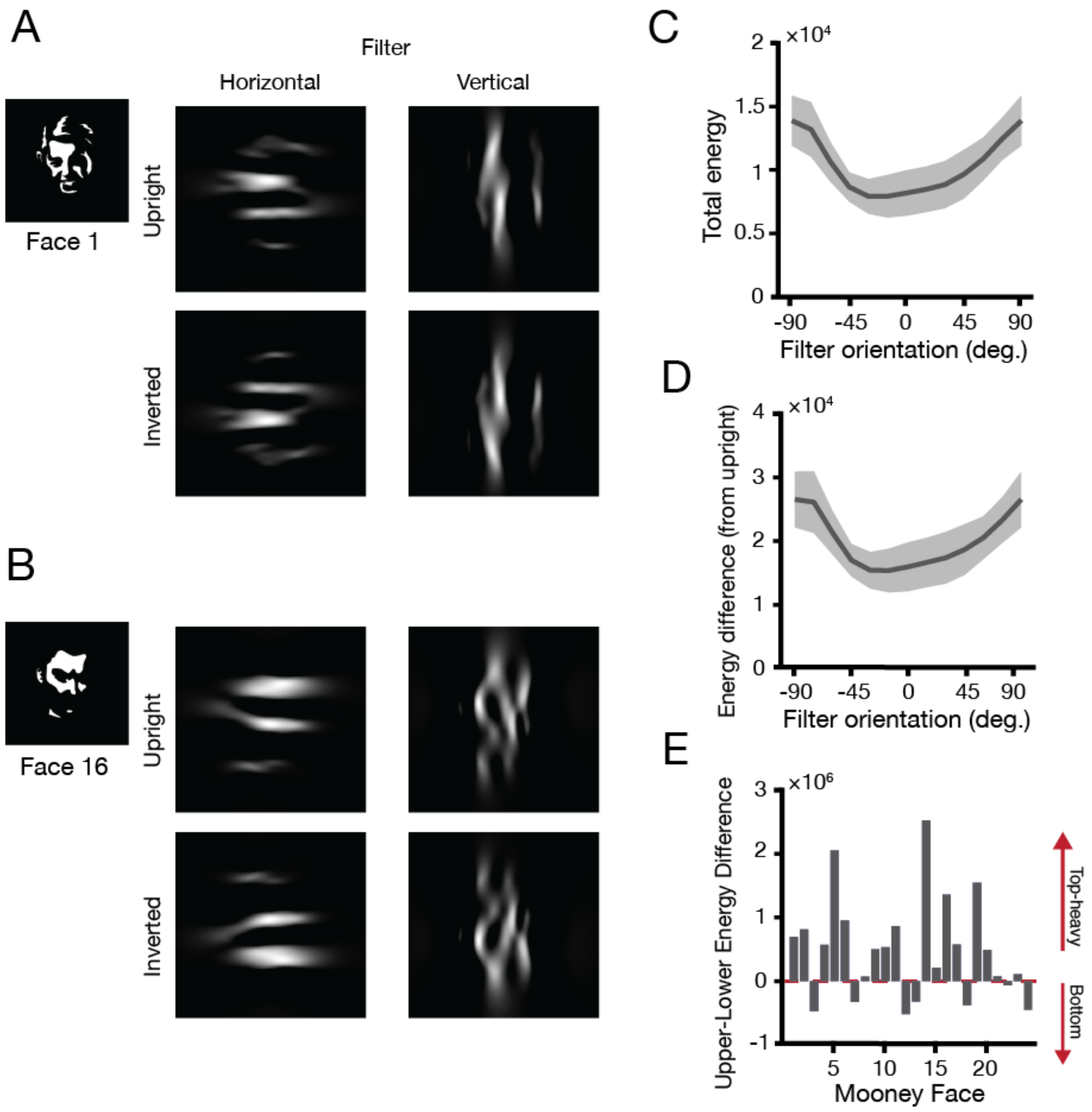

Figure S2. Image analyses for Mooney faces
(A&B). Face 1 (A) and Face 16 (B) from the Stirling Mooney database*. The first column shows the faces after horizontal filtering with a peak spatial frequency of 8 cycles per image. The second column shows the same frequency band after vertical filtering. The first row shows the results for upright Mooney faces and the second for inverted ones.
(C) The mean Fourier energy in each Mooney image, summed across spatial frequencies within each orientation band (where 0° = horizontal and 90° = vertical). Lines show the average and shaded regions show the 95% confidence interval across spatial frequencies.
(D) The mean difference in Fourier energy between upright and inverted versions of each Mooney face, plotted as a function of the orientation band (with conventions as in panel C).
(E) Differences in summed contrast energy between the upper and lower halves of each of the 24 upright Mooney faces. Positive values show 'top-heavy' and negative values show 'bottom-heavy' configurations.

many Mooney stimuli are in a rotated side profile (as in both examples in Supplementary Figure 2). The vertical content in these images is often further

*Available online at http://pics.stir.ac.uk/

emphasised by their being lit from the side. We therefore expected to see greater variability in the image content.

To examine the orientation energy within these images, the same analyses as above were performed – each image was padded with either black or white brightness values (matching the dominant image background) to extend image dimensions to 1024×1024 pixels. Each image was then fast Fourier transformed before the amplitude spectrum was multiplied with wrapped Gaussian filters with a standard deviation of 20°, centred on orientations between 0° (horizontal) and ±90° (vertical) in 15° increments. Twenty peak spatial frequencies were tested in linear steps between 1-30 cycles per image and a bandwidth of one octave. After the inverse Fourier transform, the RMS contrast of all resulting images was adjusted to match the average RMS contrast of the original image set. Images were then cropped to retain only the central 512×512 pixels to avoid edge artefacts. Inverted images were produced by mirror-reversing the image on the vertical plane. Examples are shown after horizontal and vertical filtering at 8 cycles per image for the two example faces. Notice (especially for the face in Supplementary Figure 2A) that there is a strong vertical component to the image and that the position of this energy changes after image inversion. The same is true for horizontal content, particularly for the face in Supplementary Figure 2B.

The total energy within each face was calculated by summing across all spatial frequencies. The mean value across orientation for the 24 Mooney faces examined herein is shown in Supplementary Figure 2C. Here it can be seen that the average across faces shows a bias towards the vertical orientations, unlike the dominance in horizontal bands with photographic face images. As above, this is due to the many Mooney images that have side-profile views with lighting from the side. We next characterised the differences in potential target and flanker faces that could drive the crowding of an upright target face (as observed in the study by Farzin et al.[27]). Inverted versions of each Mooney face were filtered and subtracted from upright versions, separately within each spatial frequency and orientation band (on a pixel-by-pixel basis), with the energy across the image then summed. The resulting image differences were squared and summed to compute the total energy difference between the faces. The average difference (across spatial frequency)

within each orientation band is plotted in Supplementary Figure 2D. This analysis reveals image differences between upright and inverted stimuli at all orientations, though unlike the pattern for photographic face images, this is greater in the vertical bands than in other ranges.

Finally, as with the photographic images used in Experiment 5, we also computed the extent to which each Mooney face was top- or bottom-heavy in its distribution of contrast energy. This was computed by summing the squared contrast energy in the bottom half of the image and subtracting this value from the same sum in the upper half. Results are shown in Supplementary Figure 2E for upright versions of each of the 24 faces in this set, where positive values again indicate top-heavy facial configurations. A majority of the upright Mooney faces in this set were indeed top-heavy (17/24), though some showed small biases towards being bottom-heavy. The mean value is nonetheless positive (4.82×10^5), and a one-sided t-test shows this value to be significantly different from zero ($t_{23} = 2.91$, p=.008). In other words, when they are upright, Mooney faces are on average top-heavy stimulus configurations just like photographic faces. The inversion of these stimuli would then predominantly produce bottom-heavy stimuli.

The outcome of these analyses suggests that similar processes can account for the effects of crowding on Mooney faces[27] as those that we report to account for the effects on photographic face images[26]. Although Mooney faces are certainly more difficult to recognise than photographic face images, perhaps due to the attenuation of horizontal content shown in our analyses above, it is not the case that they contain no oriented content at all. Our analyses here demonstrate that a bank of oriented filters produces outputs with a bias towards the vertical, and that the structure of these variations changes from a predominantly top-heavy configuration to one that is predominantly bottom-heavy when inverted. As in the results of our Experiment 5, this shift from top- to bottom-heavy configuration in flanker elements (relative to an upright target face) would be expected to reduce crowding, similar to the effects seen with letter-like stimuli[23]. As we argue in the General Discussion, these stimuli can certainly produce inter-feature crowding (Farzin et al.[27], Experiment 3), suggesting that these contours are themselves susceptible to crowding, just as occurs within photographic face stimuli[29]. The effects of task

difficulty are also apparent with these stimuli (Farzin et al.[27], Experiment 4), just as seen in previous studies with photographic images[26] and in Experiment 4 of our study. We therefore argue that Mooney faces do not require an additional stage of holistic crowding to account for the observed effects of flanker orientation – simpler operations based on target-flanker similarity of the kind invoked more generally to account for crowding suffice to account for the entirety of these effects.

Simulations of the effects of task difficulty on face crowding

In Experiment 2 we demonstrate that the selectivity of face crowding is identical for upright and inverted target faces when the task involves judgements of horizontal eye position. This differs from the patterns observed in Experiment 1 for judgements of identity, and in Experiment 3 with judgements of vertical eye position (Figure 3). We attribute this to the susceptibility of these latter tasks to inversion[30-32], not because this eliminates holistic processing *per se*, but because the resulting increase in task difficulty obscures the selectivity of crowding for target-flanker similarity. Accordingly, the results of Experiment 4a demonstrate that it is possible to obscure the selectivity of crowding with upright faces by increasing task difficulty. We achieved this by reducing the interocular separation in our face stimuli. Conveniently, this also presents the opportunity to conduct model simulations of this process, and to consider how this finding generalises to other tasks like identity judgements.

We thus performed a set of simulations on the effect of crowding for faces, in the context of horizontal eye judgements. This allowed us to consider both the potential mechanisms underlying these crowding effects and the effect of task difficulty. We first assume that there exists a population of detectors that is sensitive to dimensions such as eye position. This is consistent with both theoretical proposals regarding “face space”[33], adaptation effects that shift the perceived eye position within faces[34,35], and physiological measurements in the Inferior Temporal (IT) lobe of macaques[36,37]. Here we simulate a population of detectors selective for interocular eye separation in particular. We do so for the ease of modelling, rather than as a proposal that a population of this nature would be specifically utilised for this purpose – of course, interocular eye separation could be encoded either wholly by or in conjunction with cells selective for other facial properties.

Within this population, we assume that each detector is sensitive to a range of eye separations with a Gaussian tuning function that has a peak sensitivity centred on a particular eye separation, and some sensitivity to nearby values of eye separation, similar to the selectivity of V1 neurons for orientation[38], MT/V5 neurons for direction[39], and so on. Following the principles of population coding[40] the

distribution of the resulting population activity would be a Gaussian function centred on the eye separation of the stimulus, with a bandwidth of activity equivalent to the sensitivity bandwidth of the underlying detectors. The perceived value of eye separation could then be read out from this distribution (e.g. as the peak response, or via maximum likelihood estimation).

The relationship between detector sensitivity and the population response means that we can simulate the population response directly as a Gaussian function. We do so here by generating a Gaussian function with a base value of 0.1 and a peak of 1.0, using one free parameter for the standard deviation (to mimic the sensitivity bandwidth of the underlying detectors) and another for the magnitude of Gaussian noise that was added to this response distribution. If we encode the "normal" reference face with an eye-separation value of zero, then the population response to this face will be a Gaussian distribution centred on zero, as shown in Figure S3A. These responses are shown as the average of 720 trials (as in our experiments), generated with an SD of 8 pixels for illustration, and with a comparatively large range in the x-axis of ±60 pixels to make the population response clear. When the crowded target face is the same as the reference (on "target same" trials) then a similar distribution would arise for this interval. A target face with a large inwards shift would produce a similar Gaussian with a mean located at -20 pixels for the easily detected larger inwards shifts (as in Experiment 2). With this model, we can depict the task as involving a judgement regarding whether the peak of the population response lies on either side of a criterion value – depicted here as a dashed line at -10 pixels (Figure S3A). This is an ideal criterion for the 20 pixel eye shifts, sitting midway between the peak response to either face type. Peaks to the right of this criterion would be classed as the "same" as the reference; those to the left would be classed as "different". We can therefore simulate the task performed by our observers in this way.

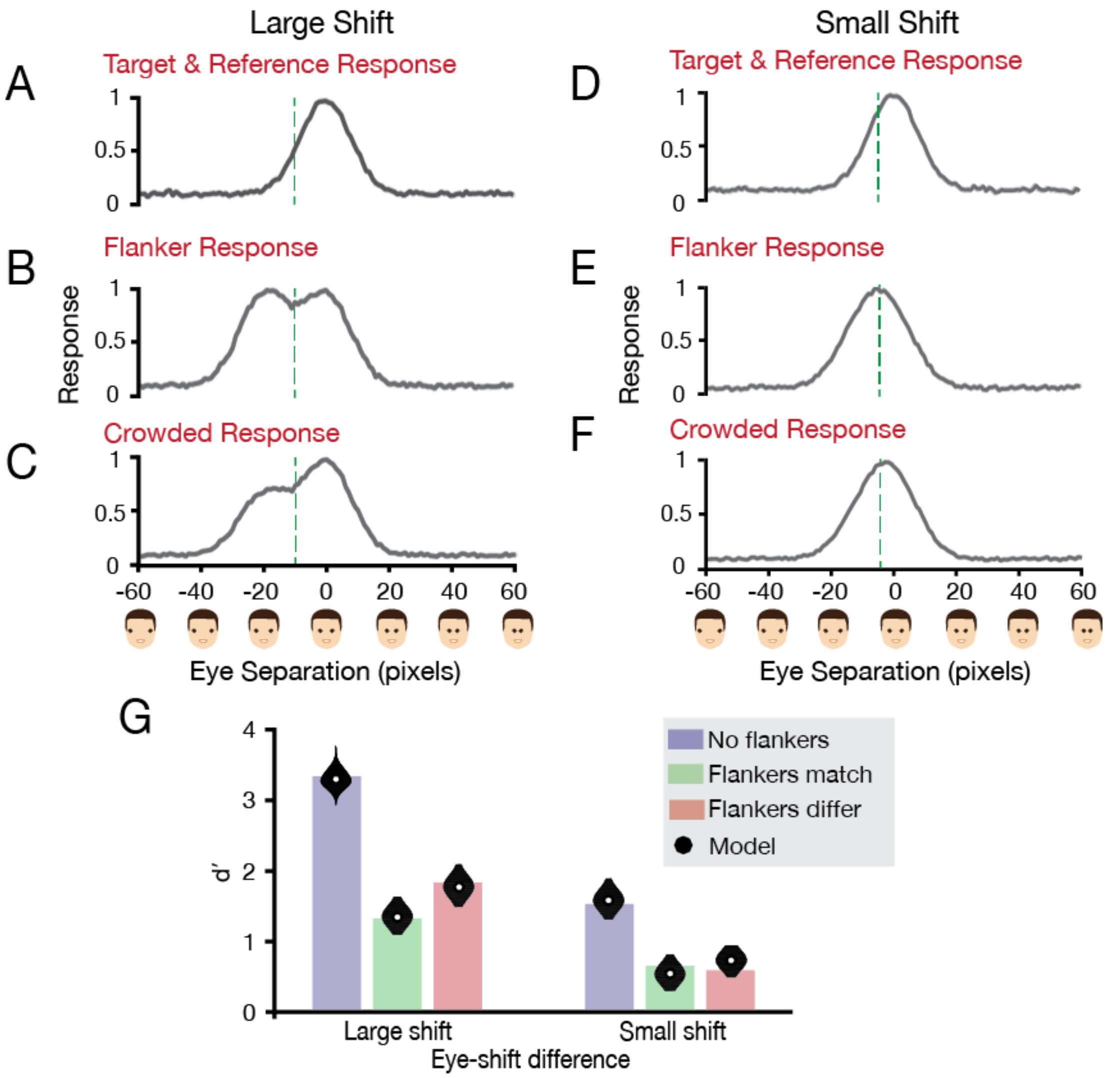

Figure S3: Model simulations for the crowding of eye positions

(A) *Reference response distribution (large shift):* Population response distributions to the reference face, with interocular eye separation on the x-axis (in pixels) and the population response on the y-axis. The green dashed line indicates the decision criterion at -10 pixels. The solid line plots the mean response across 720 trials (as in our experiments), with the shaded region showing the standard error of the mean. Responses to an unaltered target face (on "target same" trials) would be identical.
(B) *Flanker response distribution (large shift):* Simulated responses to the combination of all six flanker elements. Three flankers had an eye separation centred on zero (unaltered) and three had the eyes shifted inwards by 20 pixels (as in Experiment 2)
(C) *Crowded response distribution (large shift):* The combined population response after crowding, modelled as the weighted average of target and flanker responses shown in A and B. A target weight of 0.66 has been applied.
(D) *Reference response distribution (small shift)*: Population response distributions to the reference face with more difficult judgements. Plotted as in panel A, with the decision criterion now at -5 pixels.
(E) *Flanker response distribution (small shift)*: Simulated responses to the six flanker elements. Three flankers had zero eye separation; three had the eyes shifted inwards by 10 pixels (as in Experiment 4a and 4b).
(F) *Crowded response distribution (small shift)*: The combined population response after the weighted average of target and flanker responses in D and B with a target weight of 0.66.
(G) *A comparison of observed and simulated d' values.* Mean d' values from Experiments 2 and 3 are shown here as bars, with simulated values from the best-fitting version of our model shown via violin plots. The simulated mean d' (of 1000 simulations) for each condition is shown as a black circle.

On crowded trials, targets were surrounded by six flankers: three reference faces and three faces with eyes shifted inwards (by 20 pixels in Experiment 2). The combined population response to these flankers would be a bimodal profile with peaks at each of the two eye-separation values, as shown in Figure S3B (again the average of 720 trials). In order to simulate crowding in these instances, we follow recent models of crowding that depict the process as a pooling of target and flanker elements[41-45], and in particular to models that attribute this pooling to a combination of population responses to the target and flanker elements[42,43]. Rather than directly averaging these population profiles, we take a weighted average that allows a modulation of the precise combination of target and flanker responses, similar to previous models[41,46]. When the weighting of the target is high in this combination (relative to that of the flankers) there will be less crowding than when the weighting of the target is low. In this model, the precise weighting of the target was set as a third free parameter ranging from 0-1, with the flanker weighting equal to one minus this value. If we multiply the population response of the target by the target-weighting value, and the population response to the flankers by the flanker-weighting value, then the crowded combination is produced by adding these values.

An example crowded population profile for the large eye-shifts is plotted in Figure S3C, produced with a target weight of 0.66. This gives a bimodal response, albeit with a higher peak (on average) for the target eye separation, given the higher weighting of the target response in this "target same" trial. For this population and this value of the target weight, we observe the reverse pattern of bimodality on target-different trials when the response to the target would be centered on -20 pixels. Nonetheless, in both cases, the secondary peak in this response distribution (caused by the flankers) increases the potential noise in the population response to lead to errors on individual trials. The potential for errors is increased as the flanker weighting increases.

To obtain a release from crowding when the flankers are inverted (as in the "flankers differ" condition of our results), we can therefore simply reduce the weight of the flankers in the weighted average. This is similar to the way that prior models have simulated the effects of target-flanker similarity[46] and the reduction in

crowding with an increase in target spacing[42]. In other words, with an upright face amongst upright flankers there is a high flanker weight in the average, which is then reduced for an upright target amongst inverted flankers. The precise degree of the release from crowding is the fourth free parameter in our model, implemented as a value between 0-1 that is subtracted from the target weight in the "flankers match" condition.

Task difficulty is introduced here simply by decreasing the eye-separation to 10 pixels, as in Experiment 4a. As with the larger eye shift, the population response distribution to a "normal" reference face would be centered on zero (Figure S3D). When the target matches the reference face, the response would be identical. However, when the target face has a small inward eye shift ("target different" trials), the response would be represented by a Gaussian distribution centered at -10 pixels. The ideal criterion value would thus lie at -5 pixels, sitting midway between the peak response value to a normal face and the peak to a face with a 10 pixel inwards eye shift (dashed line, Figure S3D). The target distribution is then combined with the population response to the flankers. As in Experiment 2, three flankers had "normal" eyes and three had eyes shifted inwards, in this case by 10 pixels. Given the broad response profiles to these values, the reduction in eye shift for this experiment means that the combined response to the six flankers in the "flankers match" condition would have a unimodal profile centred on the criterion value (Figure S3E).

As seen in Figure S3F, the response profile for the combined target and flanker responses (produced with a weight of 0.66) is also unimodal, with a peak between the target value and the decision criterion. As such, there is a higher rate of responses on the side of the flankers compared to the 20 pixel eye shift, which would increase the number of errors. A similar increase in errors would be observed in "target different" trials. This illustrates that when difficulty is increased by reducing the eye shift (as in Experiment 4a), there is greater overlap between target and flanker distributions, and thus a greater propensity for errors to arise due to noise.

From the above distributions we can obtain d' values by simulating both target-same and target-different trials and extracting the peak population response on each trial. Using the location of these peaks on each trial in relation to the

decision criterion (as above), we can score each trial as producing a correct or incorrect response, and then compute a percent correct score in each condition, as in our experiments. We performed these simulations with the same number of trials as the real experiment – 720 trials per observer with 5 observers – repeated 1000 times. The best-fitting parameters for the model were an SD of 13.47 pixels, a noise magnitude of 0.32, target weighting of 0.66 (out of 1), and a crowding release of 0.09 (the difference in target weight in the "flankers match" vs. "flankers differ" conditions).

The results of these simulations are shown in Figure S3G. Mean d' values from our experiment are shown as the bar plots, with simulated distributions of d' values shown in each case as a violin plot for the distribution of all simulated values, where the mean of the 1000 simulations is a white circle. For the "easy" condition with large eye shifts, the model clearly follows the data – d' values are high when the target is uncrowded, decreased with flankers that match the upright orientation of the target, and less impaired when the flankers differ by being inverted. For the difficult condition ("small shift"), uncrowded performance drops significantly, with a further decrease for the crowded conditions. The release from crowding is then considerably muted in these simulations – because performance is reduced overall, the effect of noise is greater and the release from crowding has far less effect. The mean difference between crowded d' values for our model is 0.42 with large eye shifts and 0.20 for the more difficult small eye-shift condition. Note that the effect of task difficulty in this sense has nothing to do with our free parameters – this is simply introduced by altering the input values for eye separation from -20 to -10 in the target-different conditions. It is the combination of lowered performance and noise that flattens the selectivity of crowding for target-flanker similarity.

How might we then implement the effect of inversion within this framework? For example, for the effects we observe in Experiment 3 with vertical eye-judgements, inversion could be implemented in several ways. Inversion is generally thought to produce a shift from configural processing to more local processing[1]. This may result in either a specific impairment in configural dimensions (that are not coded in a local/featural manner) or the use of inappropriate facial landmarks. To model the effects of vertical eye-position in this sense, inversion could be

implemented as an increase in the noise associated with these eye-judgements, thereby increasing the propensity for errors, or it may broaden either the spatial or featural selectivity of the detectors sensitive to eye position (as suggested for the orientation selectivity of face identification[8], for instance). In these cases, the effects of inversion would be similar to the effect of reduced eye displacements modeled above.

Of course, inversion also disrupts the identification of faces[31,32]. These effects could similarly be modelled in a crowding paradigm via an increase in noise, an increase in the spatial or featural bandwidth of the underlying detectors, or even as a shift in the population responses towards the decision boundary. In this case, the population response would be distributed across dimensions more relevant to identity (e.g. within a “face space”[33]). Nonetheless, by expanding out from our simple model of interocular separation, we can consider that the effect of crowding on judgements of identity may arise in a similar fashion to that observed herein with judgements of eye position. Importantly however, it is not the mechanisms of crowding that would change with inversion in these cases, but rather the difficulty of the task, which in turn determines whether the selectivity of crowding for target-flanker similarity is evident or not. In this sense, we argue that crowding shares a common mechanism in all cases, rather than requiring processes specific to the holistic encoding of faces.

Supplemental References

1 Rossion, B. Picture-plane inversion leads to qualitative changes of face perception. *Acta Psychologica* **128**, 274-289 (2008).

2 Tanaka, J. W. & Farah, M. J. Parts and wholes in face recognition. *Quarterly Journal of Experimental Psychology: A Human Experimental Psychology* **46**, 225-245 (1993).

3 Young, A. W., Hellawell, D. & Hay, D. C. Configural information in face perception. *Acta Psychologica* **128** (1987).

4 Le Grand, R., Mondloch, C. J., Mauer, D. & Brent, H. P. Neuroperception: Early visual experience and face processing. *Nature* **410**, 890 (2001).

5 Diamond, R. & Carey, S. Why faces are and are not special: An effect of expertise. *Journal of Experimental Psychology: General* **115**, 107-117 (1986).

6 Thompson, P. Margaret Thatcher: A new illusion. *Perception* **9**, 483-484 (1980).

7 Dakin, S. C. & Watt, R. J. Biological "bar codes" in human faces. *Journal of Vision* **9**, 2 (2009).

8 Goffaux, V. & Greenwood, J. A. The orientation selectivity of face identification. *Scientific Reports* **6**, 34204 (2016).

9 Langner, O. *et al.* Presentation and validation of the Radboud Faces. *Cognition & Emotion* **24**, 1377-1388 (2010).

10 Viola Macchi, C., Turati, C. & Simion, F. Can a nonspecific bias towards top-heavy patterns explain newborns' face preference? *Psychological Science* **15**, 379-383 (2004).

11 Simion, F., Valenza, E., Macchi Cassia, V., Turati, C. & Umiltà, C. A. Newborn's preference for up-down asymmetrical configurations. *Developmental Science* **5**, 427-434 (2002).

12 de Heering, A. *et al.* Three-month-old infants' sensitivity to horizontal information within faces. *Developmental Psychobiology* **58**, 536-542 (2016).

13 Glen, J. C. & Dakin, S. C. Orientation-crowding within contours. *Journal of Vision* **13**, 1-11 (2013).

14 Põder, E. Effect of colour pop-out on the recognition of letters in crowding conditions. *Psychological Research* **71**, 641-645 (2007).

15 Kennedy, G. J. & Whitaker, D. The chromatic selectivity of visual crowding. *Journal of Vision* **10**, 15 (2010).

16 Kooi, F. L., Toet, A., Tripathy, S. P. & Levi, D. M. The effect of similarity and duration on spatial interaction in peripheral vision. *Spatial Vision* **8**, 255-279 (1994).

17 Gheri, C., Morgan, M. J. & Solomon, J. A. The relationship between search efficiency and crowding. *Perception* **36**, 1779-1787 (2007).

18 Chung, S. T., Levi, D. M. & Legge, G. E. Spatial frequency and contrast properties of crowding. *Vision Research* **41**, 1833 - 1850 (2001).

19 Andriessen, J. J. & Bouma, H. Eccentric vision: Adverse interactions between line segments. *Vision Research* **16**, 71-78 (1976).

20 Wilkinson, F., Wilson, H. R. & Ellemberg, D. Lateral interactions in peripherally viewed texture arrays. *Journal of the Optical Society of America A* **14**, 2057-2068 (1997).

21 Hariharan, S., Levi, D. M. & Klein, S. A. "Crowding" in normal and amblyopic vision assessed with Gaussian and Gabor C's. *Vision Research* **45**, 617-633 (2005).
22 Levi, D. M., Hariharan, S. & Klein, S. A. Suppressive and facilitatory spatial interactions in peripheral vision: Peripheral crowding is neither size invariant or simple contrast masking. *Journal of Vision* **2**, 3 (2002).
23 Dakin, S. C., Cass, J., Greenwood, J. A. & Bex, P. J. Probabilistic, positional averaging predicts object-level crowding effects with letter-like stimuli. *J Vis* **10**, 14, doi:10.1167/10.10.14 (2010).
24 Dakin, S. C. & Watt, R. J. Biological "bar codes" in human faces. *Journal of Vision* **9**, 1-10 (2009).
25 Goffaux, V. & Dakin, S. C. Horizontal information drives the behavioural signatures of face processing. *Frontiers in Psychology* **1**, 143 (2010).
26 Louie, E. G., Bressler, D. W. & Whitney, D. Holistic crowding: Selective interference between configural representations of faces in crowded scenes. *Journal of Vision* **7**, 24-24 (2007).
27 Farzin, F., Rivera, S. M. & Whitney, D. Holistic crowding of Mooney faces. *Journal of Vision* **9**, 1-15 (2009).
28 Mooney, C. Age in the development of closure ability in children. *Canadian Journal of Psychology* **11**, 219-226 (1957).
29 Martelli, M., Majaj, N. J. & Pelli, D. G. Are faces processed like words? A diagnostic test for recognition by parts. *Journal of Vision* **5**, 58-70 (2005).
30 Goffaux, V. & Rossion, B. Face inversion disproportionately impairs the perception of vertical but not horizontal relations between features. *Journal of Experimental Psychology: Human Perception & Performance* **33**, 995-1001 (2007).
31 Yin, R. K. Looking at upside-down faces. *Journal of Experimental Psychology* **81**, 141-145 (1969).
32 McKone, E. Isolating the special component of face recognition: Peripheral identification and a Mooney face. *Journal of Experimental Psychology: Learning, Memory, and Cognition* **30**, 181-197 (2004).
33 Valentine, T. A unified account of the effects of distinctiveness, inversion, and race in face recognition. *The Quarterly Journal of Experimental Psychology* **43**, 161-204 (1991).
34 Robbins, R., McKone, E. & Edwards, M. Aftereffects for face attributes with different natural variability: Adapter position effects and neural models. *Journal of Experimental Psychology: Human Perception & Performance* **33**, 570-592 (2007).
35 Susilo, T., McKone, E. & Edwards, M. What shape are the neural response functions underlying opponent coding in face space? A psychophysical investigation. *Vision Research* **50**, 300-314 (2010).
36 Freiwald, W. A., Tsao, D. Y. & Livingstone, M. S. A face feature space in the macaque temporal lobe. *Nature Neuroscience* **12**, 1187-1196 (2009).
37 Chang, L. & Tsao, D. Y. The code for facial identity in the primate brain. *Cell* **169**, 1013-1028 (2017).

38 Schiller, P. H., Finlay, B. L. & Volman, S. F. Quantitative studies of single-cell properties in monkey striate cortex ||. Orientation specificity and ocular dominance. *Journal of Neurophysiology* **38**, 1320-1333 (1976).

39 Felleman, D. J. & Kaas, J. H. Receptive-field properties of neurons in middle temporal visual area (MT) of owl monkeys. *Journal of Neurophysiology* **52**, 488-513 (1984).

40 Pouget, A., Dayan, P. & Zemel, R. Information processing with population codes. *Nature Reviews Neuroscience* **1** (2000).

41 Greenwood, J. A., Bex, P. J. & Dakin, S. C. Positional averaging explains crowding with letter-like stimuli. *Proceedings of the National Academy of Sciences of the United States of America* **106**, 12130-13135 (2009).

42 Harrison, W. J. & Bex, P. J. A unifying model of orientation crowding in peripheral vision. *Current Biology* **25**, 3213-3219 (2015).

43 van den Berg, R., Roerdink, J. B. T. M. & Cornelissen, F. W. A neurophysiologically plausible population code model for feature integration explains visual crowding. *PLoS Computational Biology* **6**, e1000646 (2010).

44 Parkes, L., Lund, J., Angelluci, A., Solomon, J. A. & Morgan, M. Compulsory averaging of crowded orientation signals in human vision. *Nature Neuroscience* **4**, 6 (2001).

45 Freeman, J., Chakravarthi, R. & Pelli, G. D. Substitution and pooling in crowding. *Attention, Perception, & Psychophysics* **74**, 379-396 (2012).

46 Greenwood, J. A., Bex, P. J. & Dakin, S. C. Crowding follows the binding of relative position and orientation. *Journal of Vision* **12**, 1-20 (2012).
